# Supplementary figures and images for: Mutation bias alters the distribution of fitness effects of mutations
Source: PLoS Biol. 2025 Jul 14;23(7):e3003282. doi: 10.1371/journal.pbio.3003282 (PMC12273949; doi:10.1371/journal.pbio.3003282)

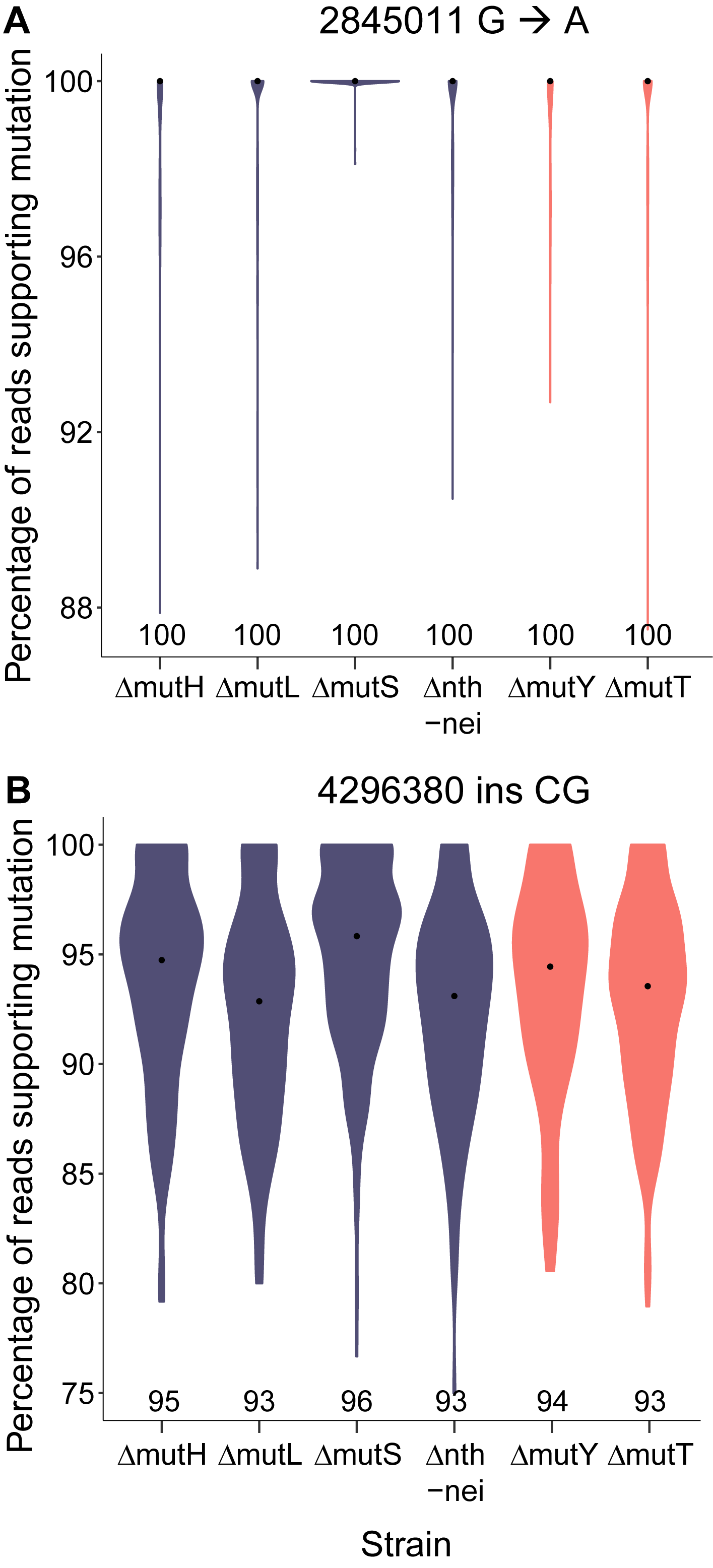

Supplement: S1 Fig — We tested whether mutations in the background of the MG1655 clone used to construct all mutator ancestors were recovered in all evolved clones, as expected if sequencing was perfectly accurate. Violin plots show the frequency of two background mutations in our WT ancestor (compared to the NCBI reference sequence NC_000913.2) in all re-sequenced MA-evolved clones (A) A G→A mutation at position 2854011, and (B) An insertion of CG at position 4296830, in evolved mutator MA lines. Values under each violin are the median of the distribution. Data underlying this figure are given in S9 Data. (TIF) [file pbio.3003282.s001.tif]

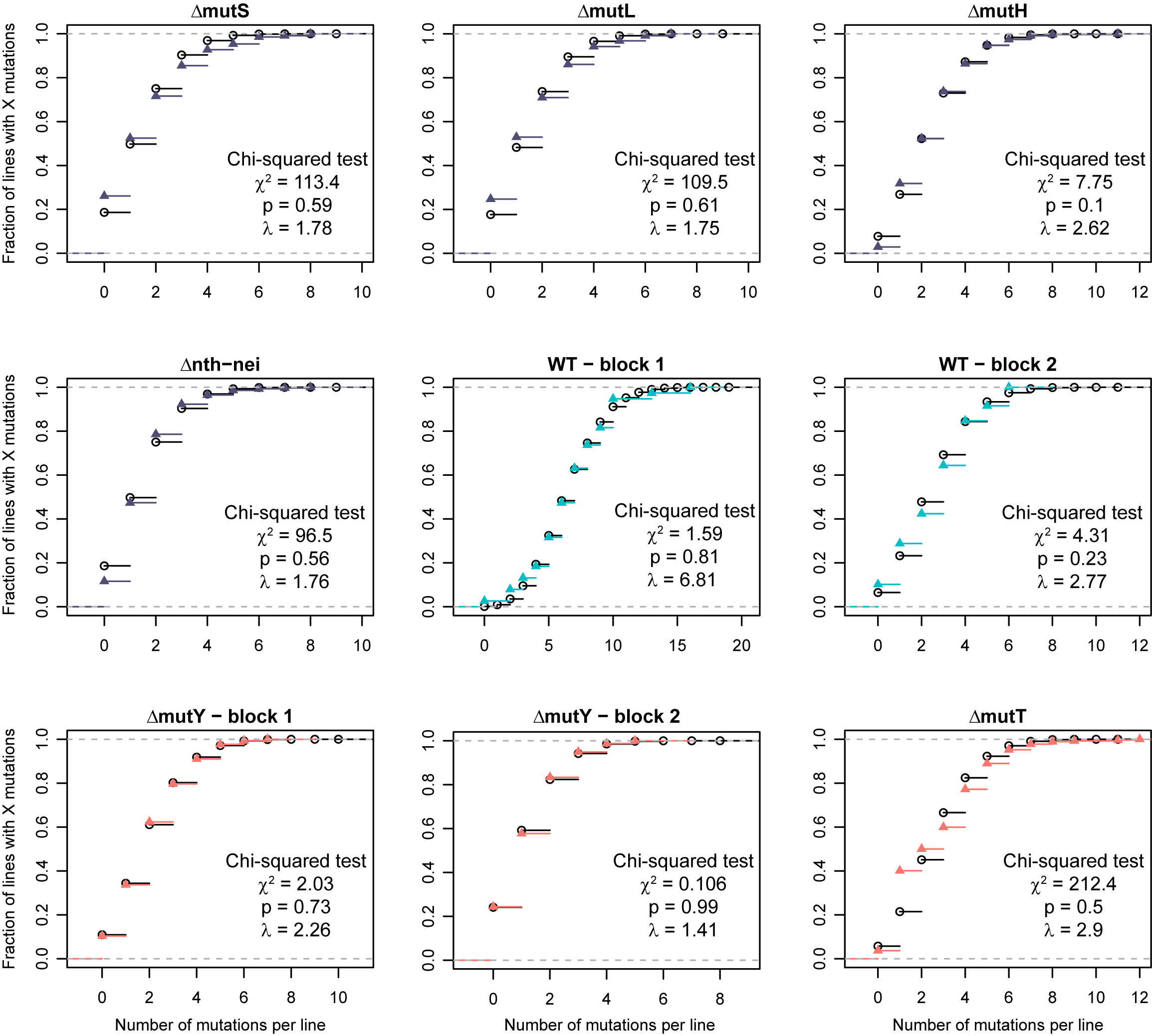

Supplement: S2 Fig — In each panel, open circles represent the expected number of mutations per MA line, assuming a Poisson distribution with λ = mean number of mutations observed per MA line. Filled triangles show the observed number of mutations per MA line. Results of a goodness-of-fit chi-squared test comparing observed versus expected distributions are given in each panel. When different MA blocks differed in the number of generations evolved (in the case of WT and ∆mutY), and therefore had significantly different mean numbers of mutations per MA line across blocks, we analyzed blocks separately. Data underlying this figure are given in S10 Data. (TIF) [file pbio.3003282.s002.tif]

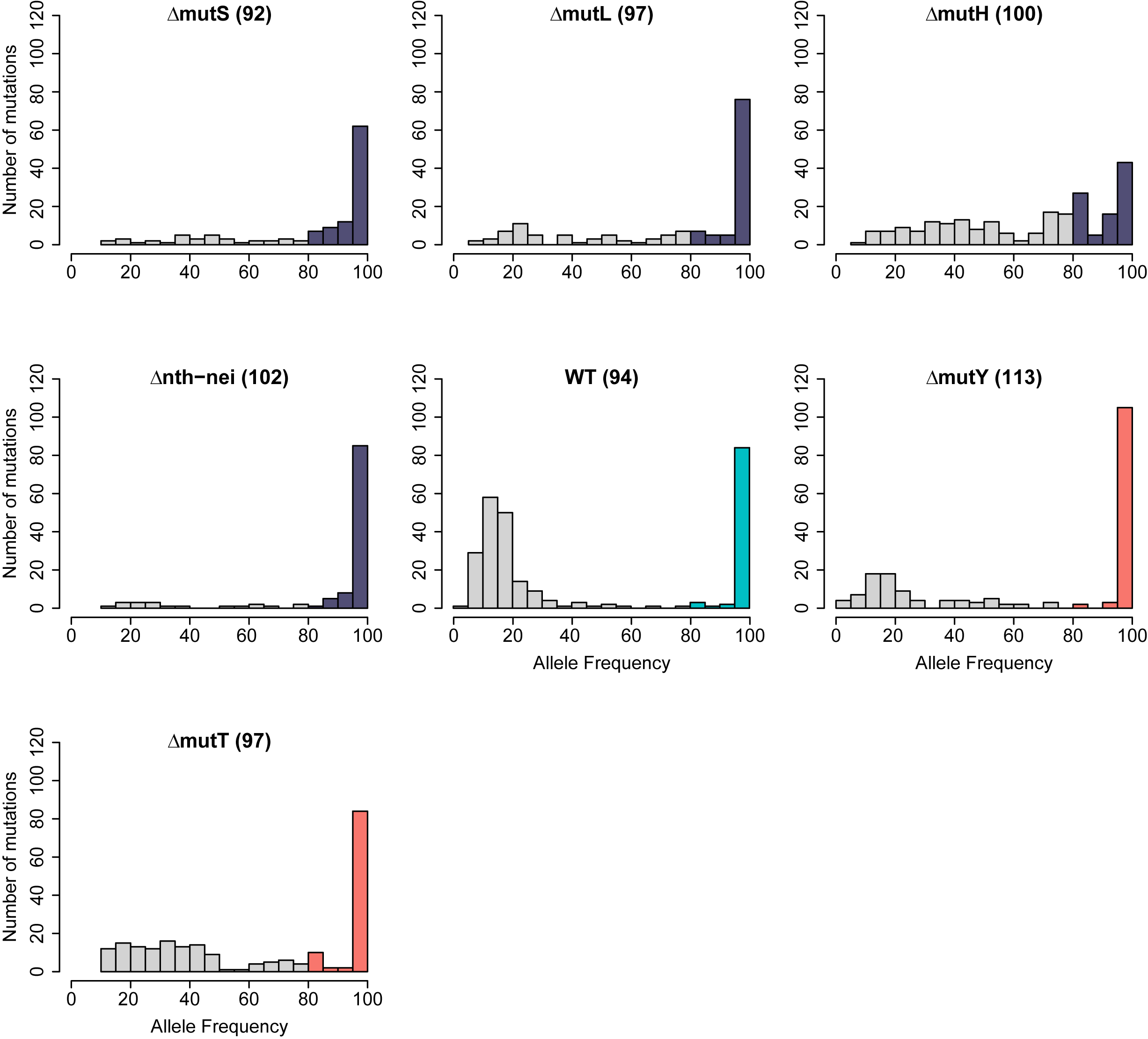

Supplement: S3 Fig — Histograms show allele frequencies of mutations in MA lines included in the single mutation DFEs. In each panel, data are pooled for all MA lines included in the DFE measurements for that strain (number of lines is given in parentheses; in these MA lines, we recovered only one mutation of >80% frequency). Gray bars represent mutations segregating in MA lines at lower frequencies (<80%) and colored bars represent mutations at >80% frequency. Data underlying this figure are given in S11 Data. (TIF) [file pbio.3003282.s003.tif]

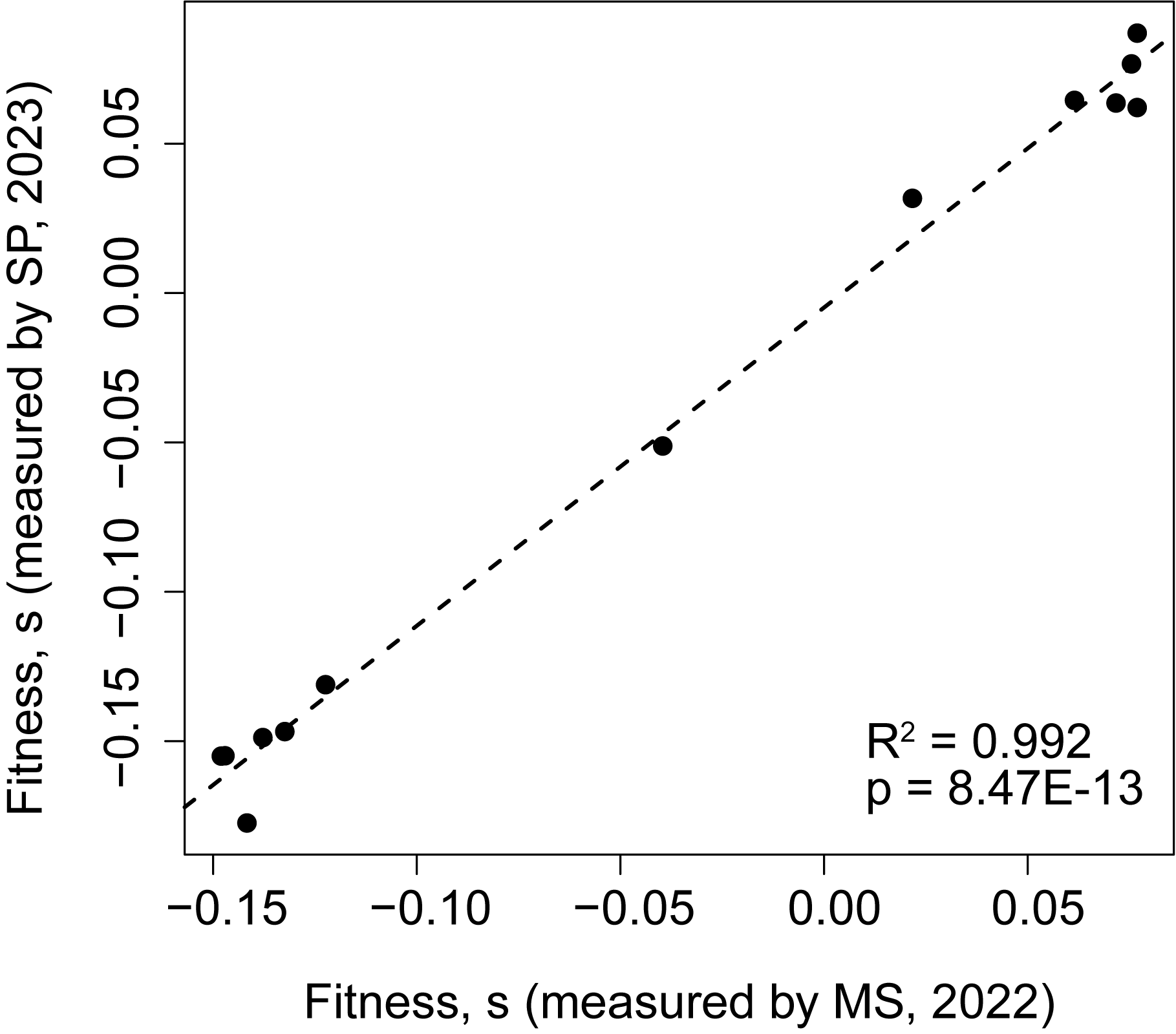

Supplement: S4 Fig — The plot shows a fitted linear regression (dashed line) and associated statistics of the relationship between fitness measurements in Glucose conducted in 96-well plates by two different experimenters in two different years, for a set of 12 WT MA clones carrying single mutations. Data underlying this figure are given in S12 Data. (TIF) [file pbio.3003282.s004.tif]

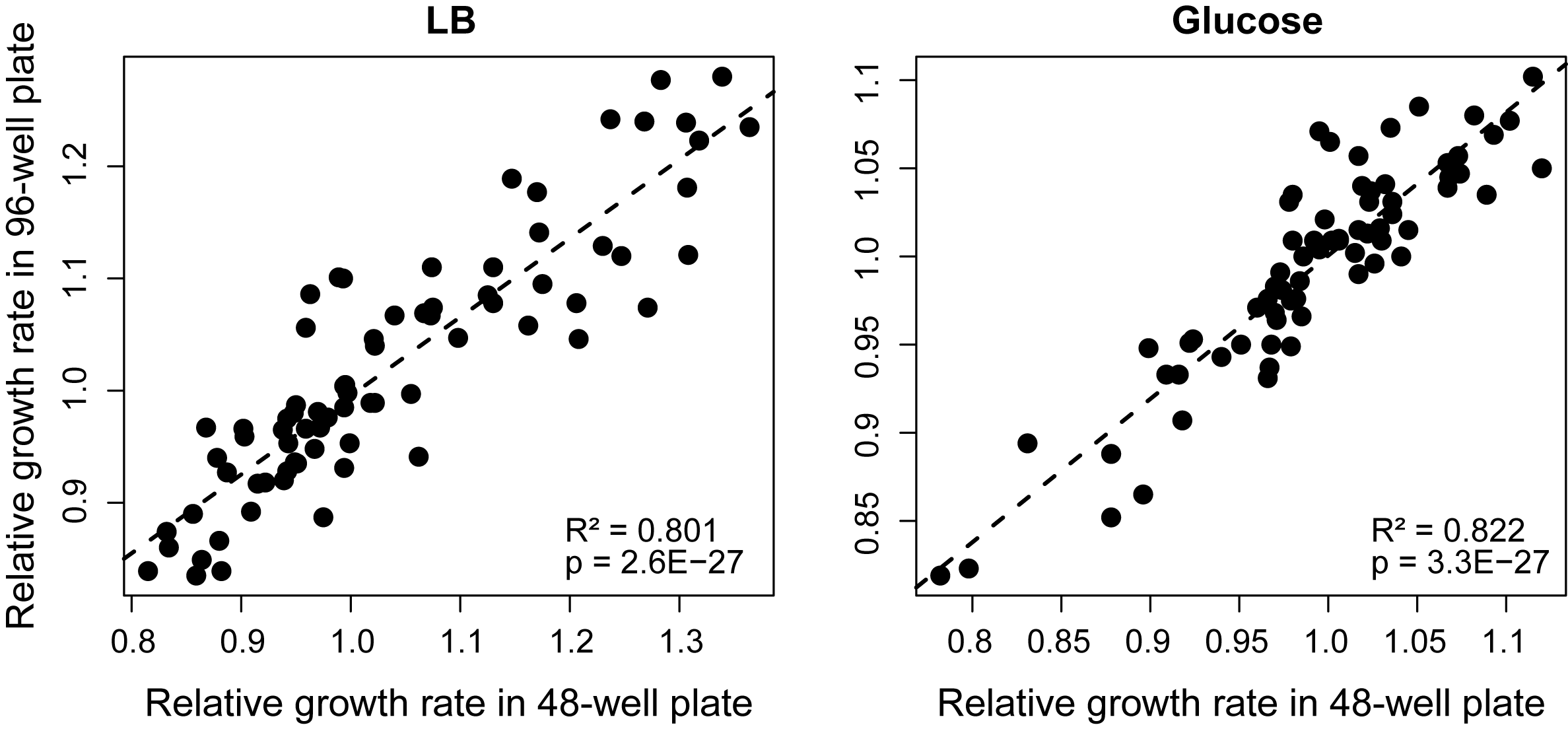

Supplement: S5 Fig — Each panel shows the fitted linear regression (dashed line) and associated statistics of the relationship between relative growth rates of 80 WT clones carrying single mutations obtained in 48-well plates (data from [1]) and 96-well plates (this study), in LB and Glucose. Data underlying this figure are given in S13 Data. (TIF) [file pbio.3003282.s005.tif]

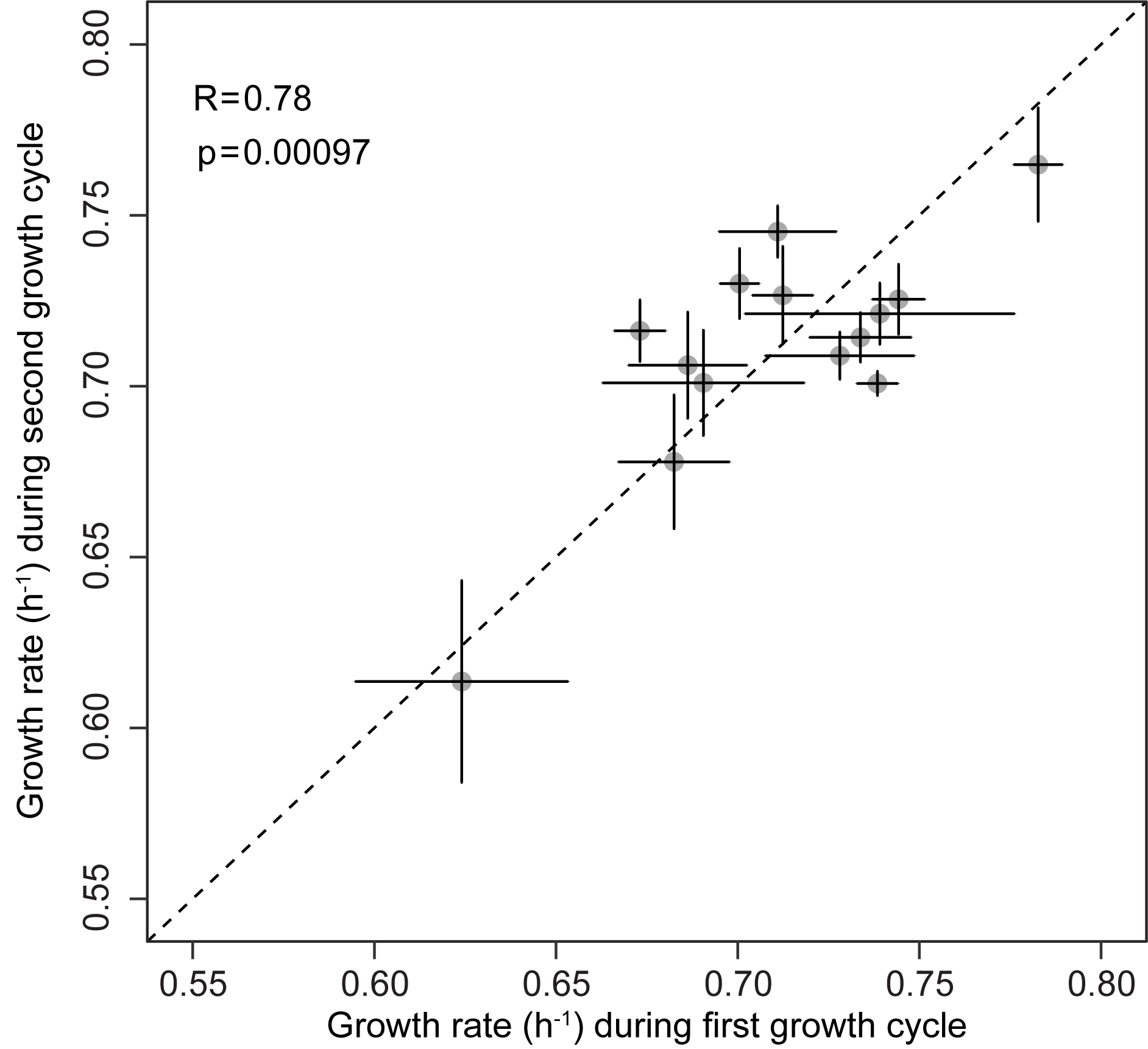

Supplement: S6 Fig — Heritable, “real” mutations identified during resequencing should have consistent effects across successive growth cycles. The plot shows growth rates of different MA clones in glucose (mean ± standard error). We show growth rates in the first 16-h growth after reviving from frozen glycerol stocks (x-axis) vs. a second 16-h growth cycle initiated using cultures from the first growth cycle (y-axis). The dashed line indicates equivalent growth rates in both cycles. Pearson’s correlation coefficient and associated p-value are shown. Data underlying this figure are given in S14 Data. (TIF) [file pbio.3003282.s006.tif]

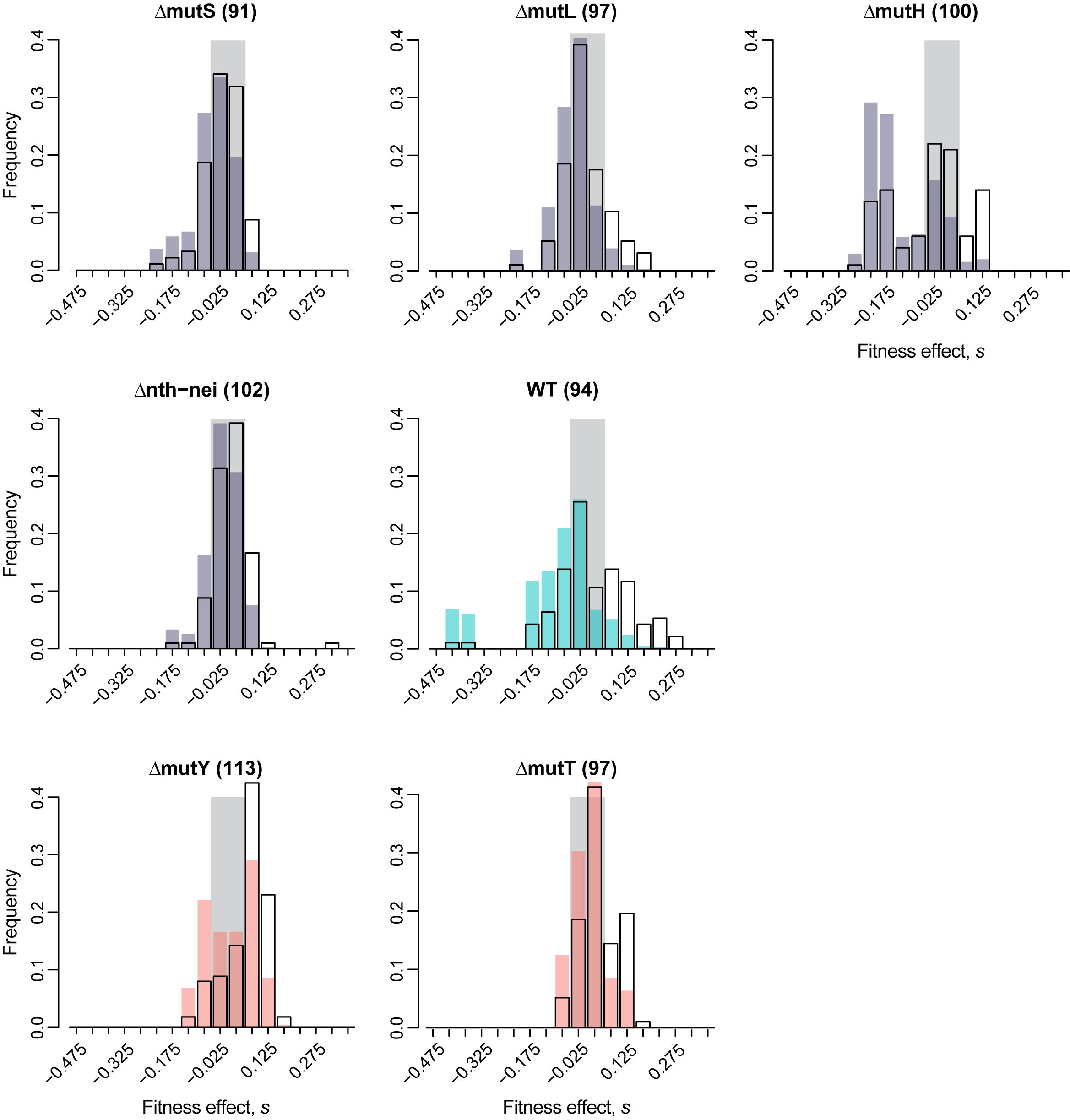

Supplement: S7 Fig — Raw (open bars) and corrected DFEs (filled bars) of single mutations in each strain’s MA-accumulated mutations tested in LB. Corrected DFEs are colored as in Fig 2. Gray areas indicate neutral mutations (s = 0 ± 0.05 to account for experimental measurement error). Data underlying this figure are given in S15 Data. (TIF) [file pbio.3003282.s007.tif]

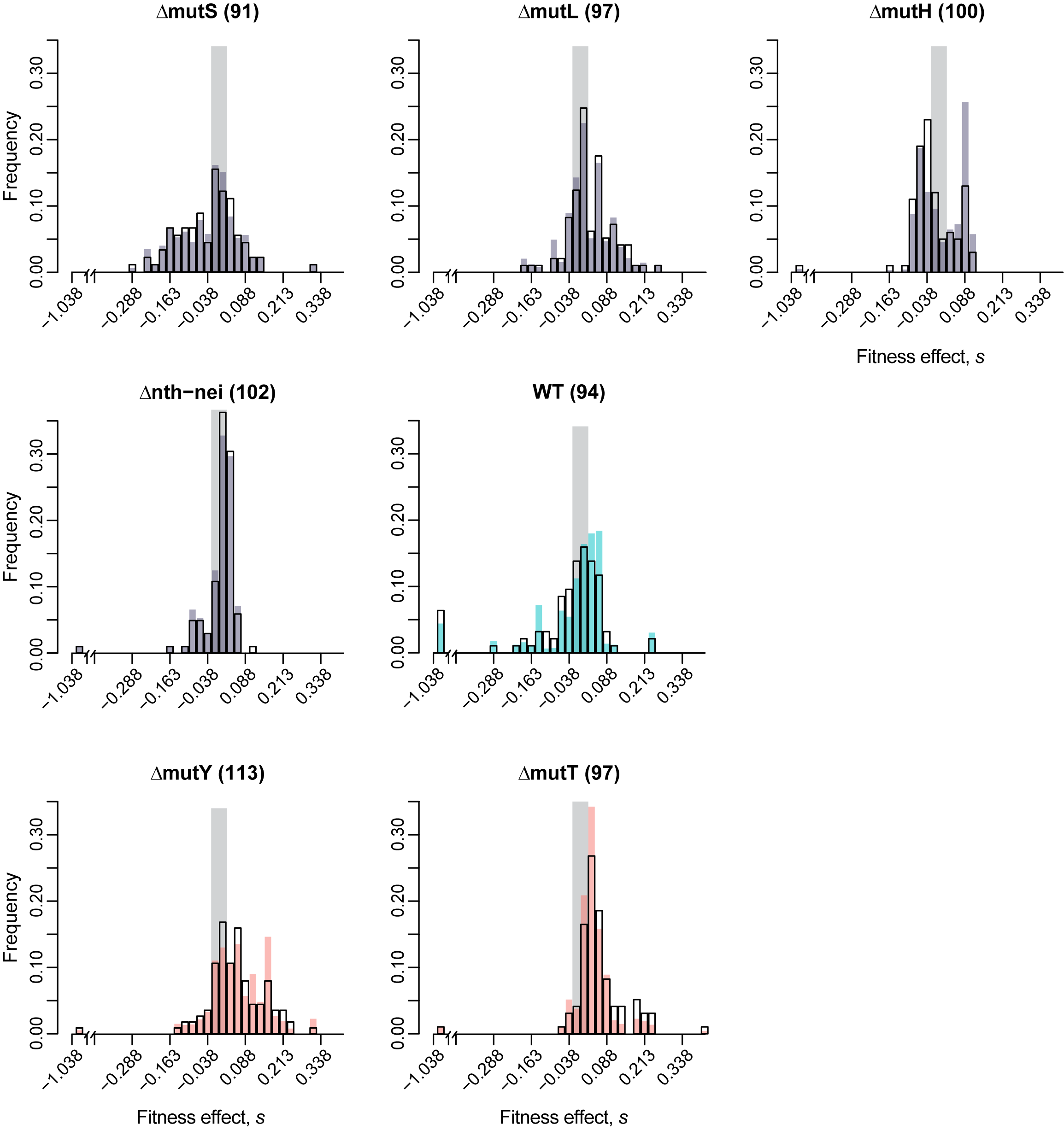

Supplement: S8 Fig — Raw (open bars) and corrected DFEs (filled bars) of single mutations in each strain’s MA-accumulated mutations. Corrected DFEs are colored as in Fig 2. Gray areas indicate neutral mutations (s = 0 ± 0.025 to account for experimental measurement error). Data underlying this figure are given in S16 Data. (TIF) [file pbio.3003282.s008.tif]

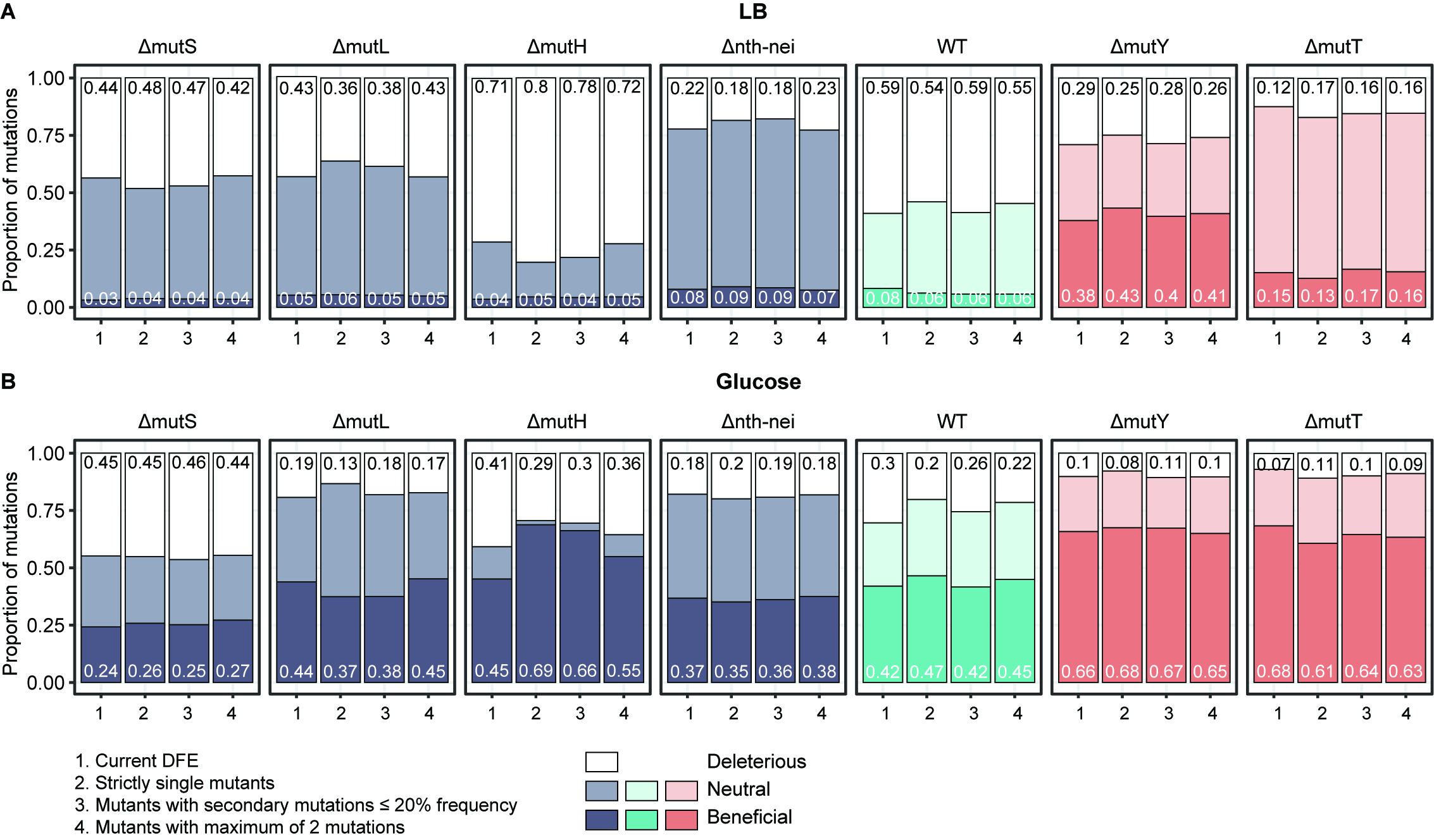

Supplement: S9 Fig — The fraction of beneficial, neutral, and deleterious mutations for DFEs constructed from MA-evolved clones filtered based on the presence and frequency of secondary mutations. We applied three sets of filters to clones from each strain, comparing each DFE (after correcting for selection bias during MA) with the original (“current”) DFE reported in Fig 4 (1): clones with exactly one mutation and no detectable secondary mutation, even at low frequency (2); clones with a secondary mutation at less than 20% allele frequency (3); and clones with exactly two mutations at any frequency (4). The proportion of beneficial and deleterious mutations is given in each bar. In all cases, chi-squared tests comparing each filtered set of clones with the current DFE, with Benjamini–Hochberg correction for multiple comparisons, showed a lack of significant differences in each case (p > 0.05). Data underlying this figure are given in S17 Data. (TIF) [file pbio.3003282.s009.tif]

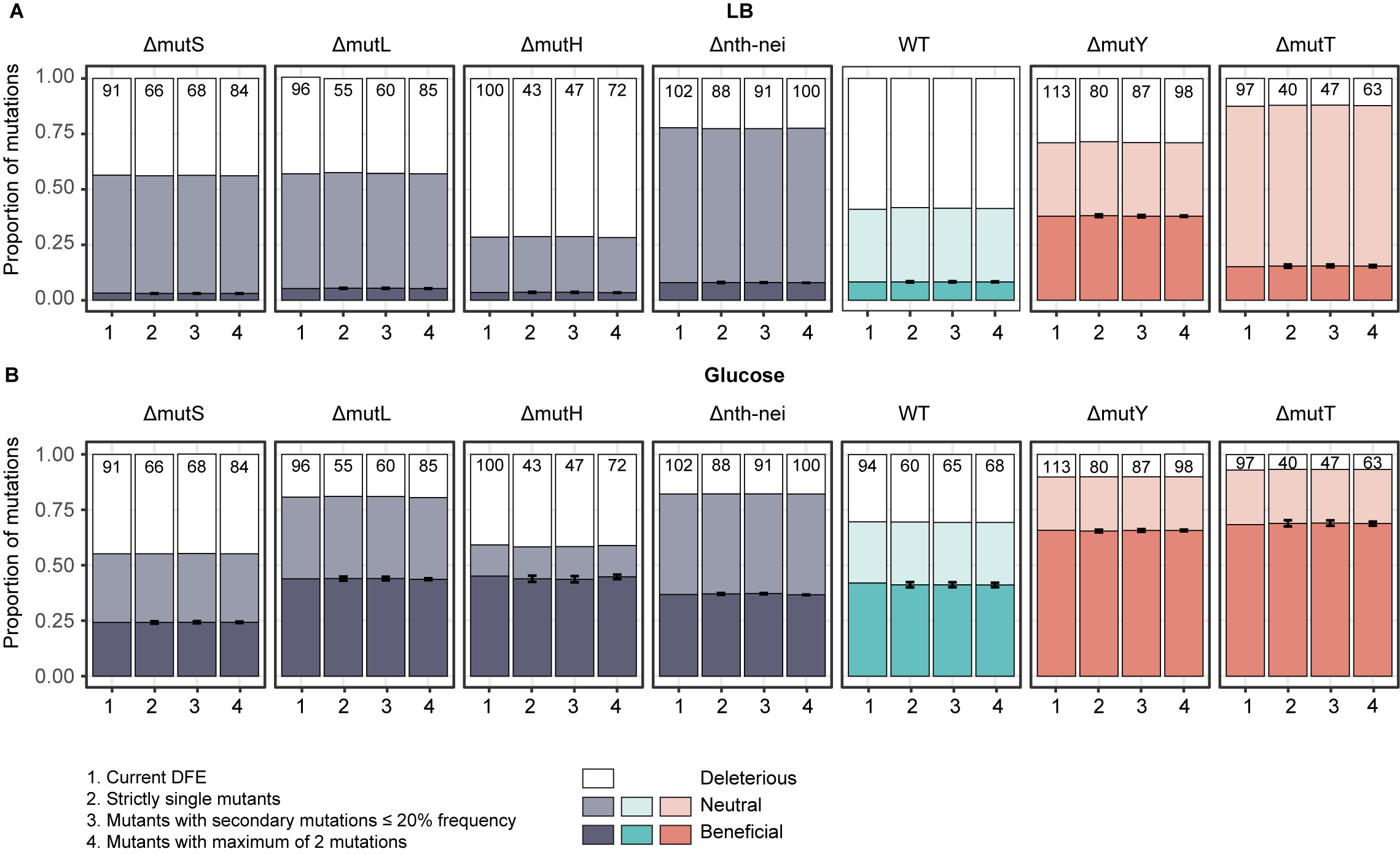

Supplement: S10 Fig — The stringent filtering described in S9 Fig reduced the number of mutants used to construct each DFE. To test the effect of reduced sample size, we subsampled the current DFE (1) reported in Fig 4 with the respective sample size of each filtered category of clones shown in S9 Fig. Plots show the results from 100 iterations, with 95% confidence intervals indicated for the beneficial fraction. The sample size (number of clones) is indicated in each bar. Chi-squared tests comparing each filtered set of clones with the current DFE, with Benjamini–Hochberg correction for multiple comparisons, showed a lack of significant differences (p > 0.05). Data underlying this figure are given in S18 Data. (TIF) [file pbio.3003282.s010.tif]

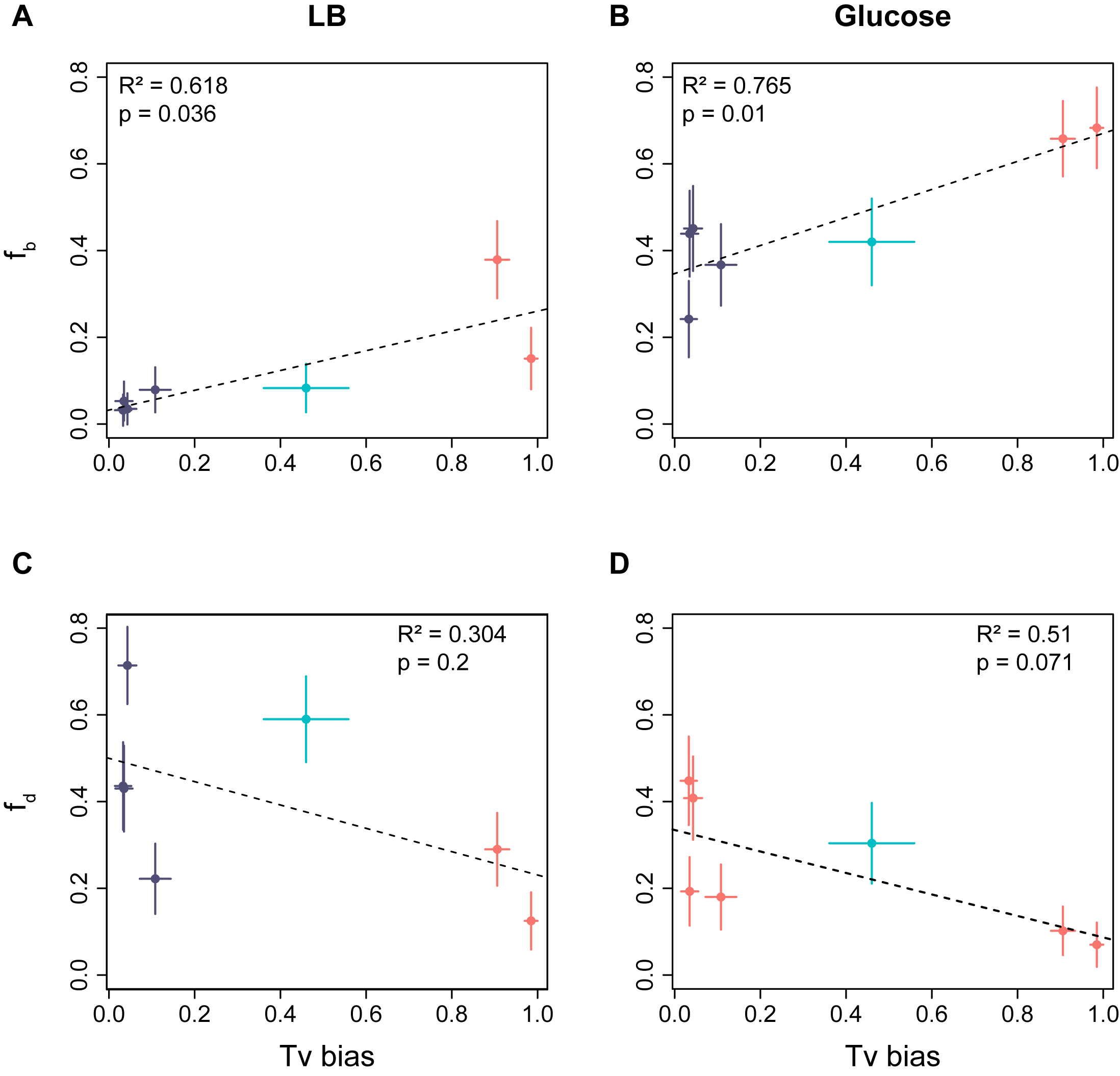

Supplement: S11 Fig — Each panel shows the linear regression fit (dashed line) and associated statistics of the relationship between Tv bias and (A, B) fb or (C, D) fd, in LB (left panels) and glucose (right panels). fb and fd values are given in Fig 3A and Tv bias values are given in Table 1. Points are colored as indicated in Fig 2. Error bars represent 95% confidence intervals around the mean. Data underlying this figure are given in S19 Data. (TIF) [file pbio.3003282.s011.tif]

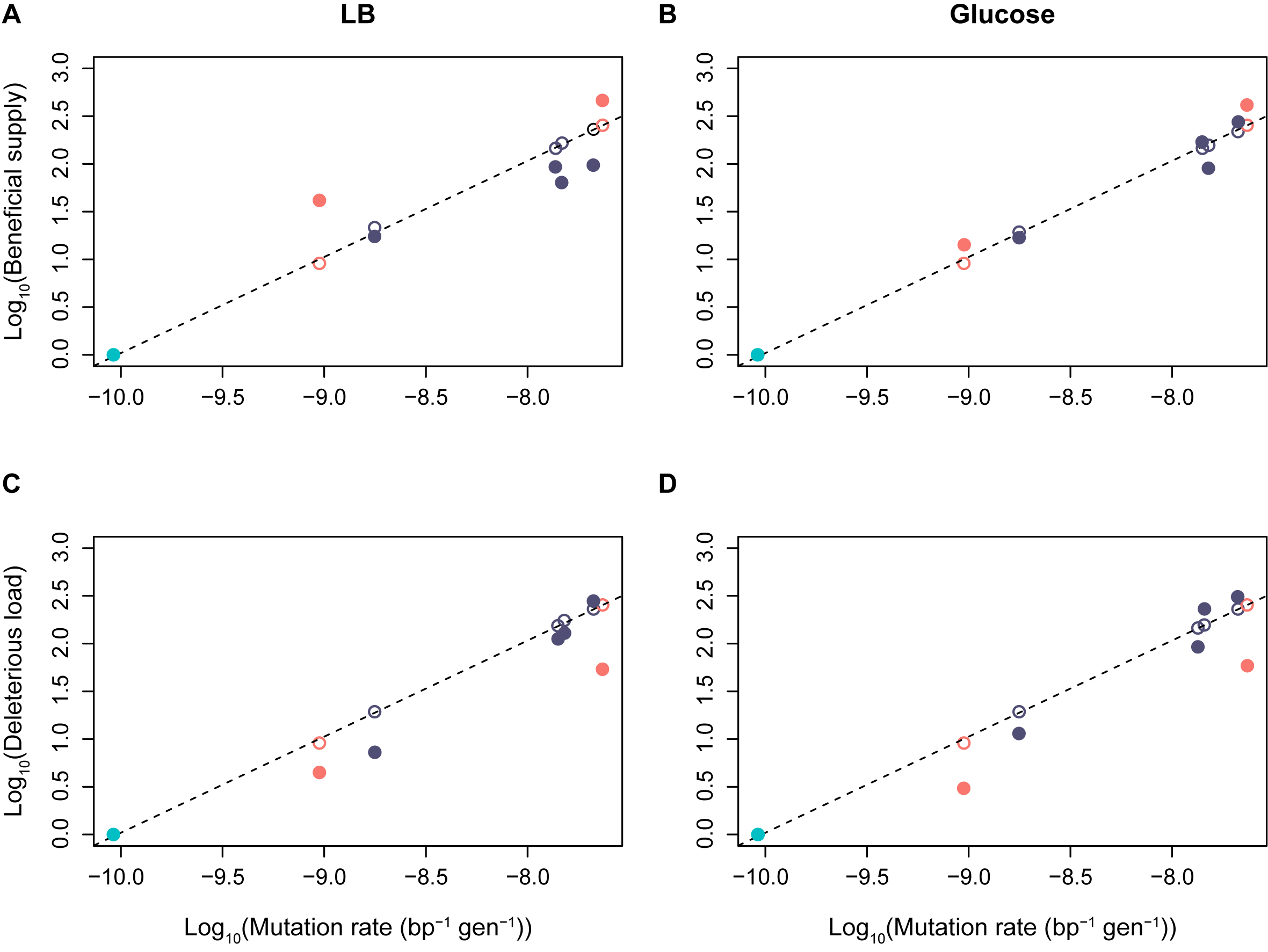

Supplement: S12 Fig — Plots show the (A, B) beneficial supply and (C, D) deleterious load experienced by the different mutators as a function of mutation rate, in LB (left panels) and glucose (right panels). Strains are colored as in Fig 1 (purple: Ts-biased strains, teal: WT, pink: Tv-biased strains). Filled circles represent supply or load calculated using the fb values obtained from the observed DFE for each mutator (Fig 4A; Sb and Ld values shown in S4 and S5 Tables, respectively); open circles represent supply or load calculated assuming fb values derived from the WT DFE (i.e., if all strains had the same DFE; Sb(WT DFE) and Ld(WT DFE) in S4 and S5 Tables, respectively). For each strain, mutation rates used for the calculations are given in Table 1. Calculations of beneficial supply and deleterious load are shown in S4 and S5 Tables. Dashed lines represent the best fit linear regression for open circles (i.e., supply or load as a function of mutation rate, assuming identical DFEs). Data underlying this figure are given in S20 Data. (TIF) [file pbio.3003282.s012.tif]

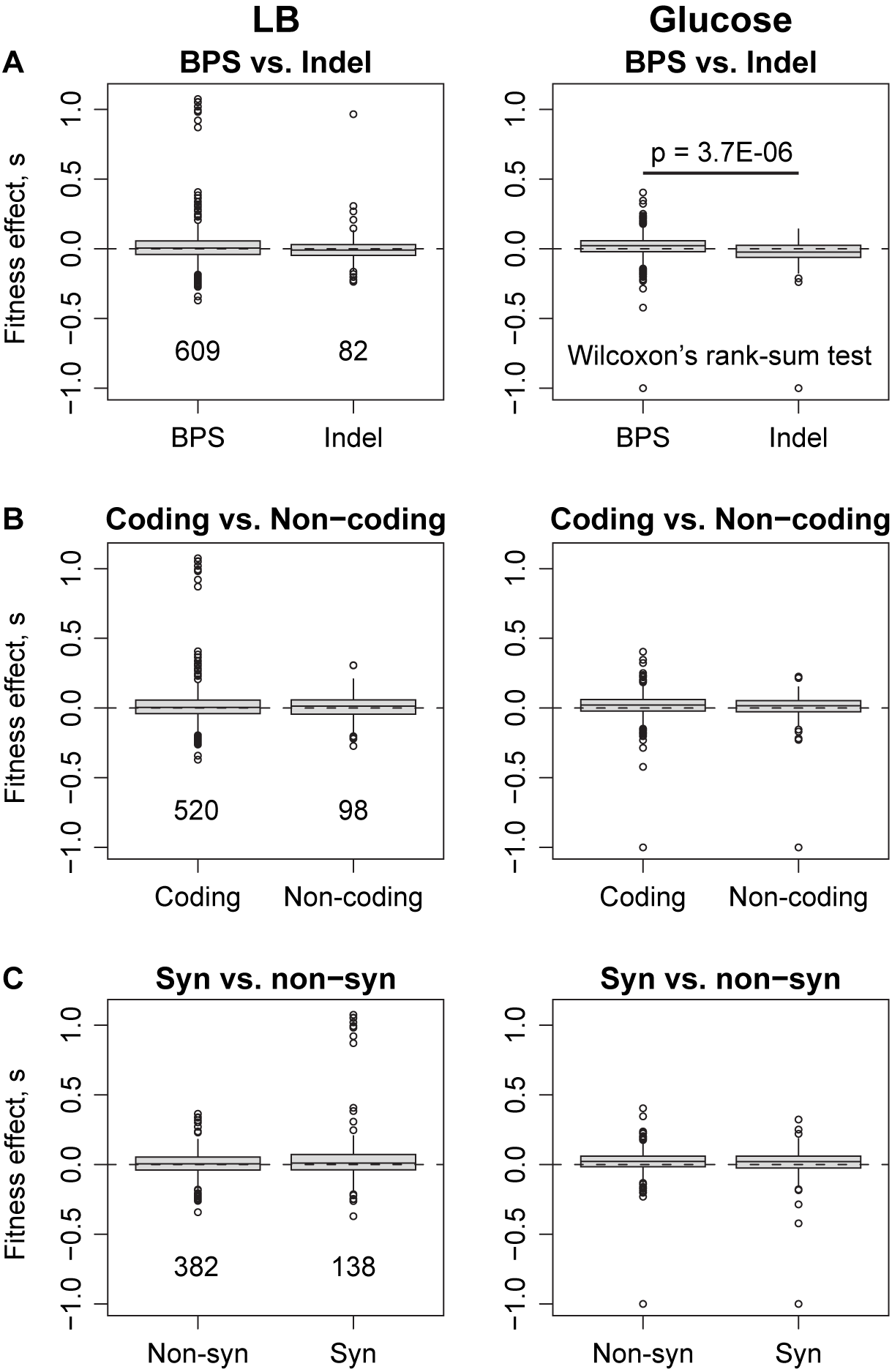

Supplement: S13 Fig — Fitness effects of (A) BPS vs. Indel mutations, (B) Coding vs. non-coding mutations, and (C) Synonymous vs. non-synonymous mutations. In each plot, data are pooled across all strains; sample sizes (total number of single mutations tested) are shown in the LB (left) panels. When differences are significant (Wilcoxon’s rank-sum tests), P-values are given in the appropriate panel. Data underlying this figure are given in S21 Data. (TIF) [file pbio.3003282.s013.tif]

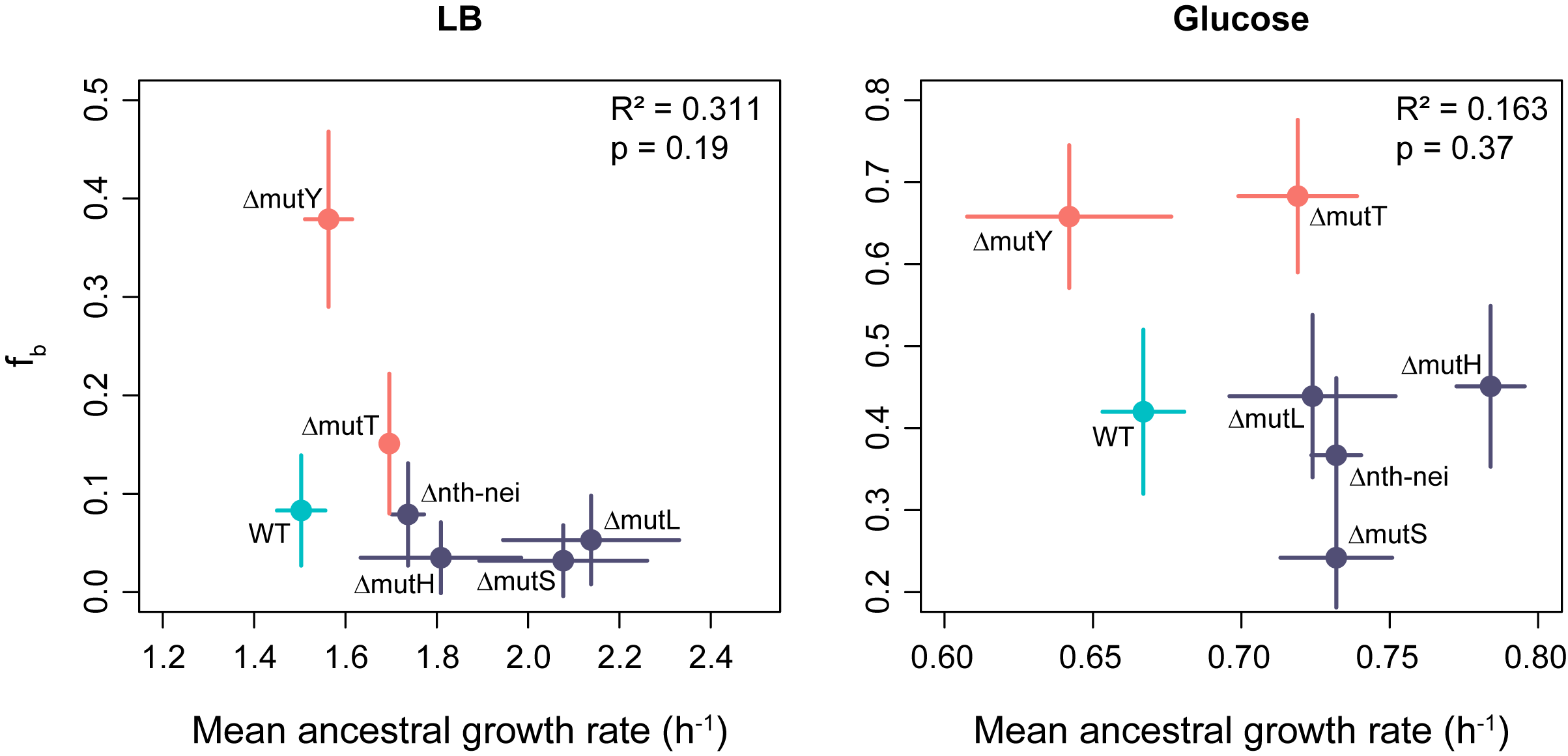

Supplement: S14 Fig — Plots show the relationship between fb and mean ancestral growth rates in LB (left) and glucose (right). Horizontal error bars represent variation in ancestral growth rates (mean ± SE) and vertical error bars represent uncertainty in fb estimates (fb ± 95% CI). The R2 and p-values from a linear regression of fb ~ mean ancestral growth rate are shown in each panel. Data underlying this figure are given in S22 Data. (TIF) [file pbio.3003282.s014.tif]

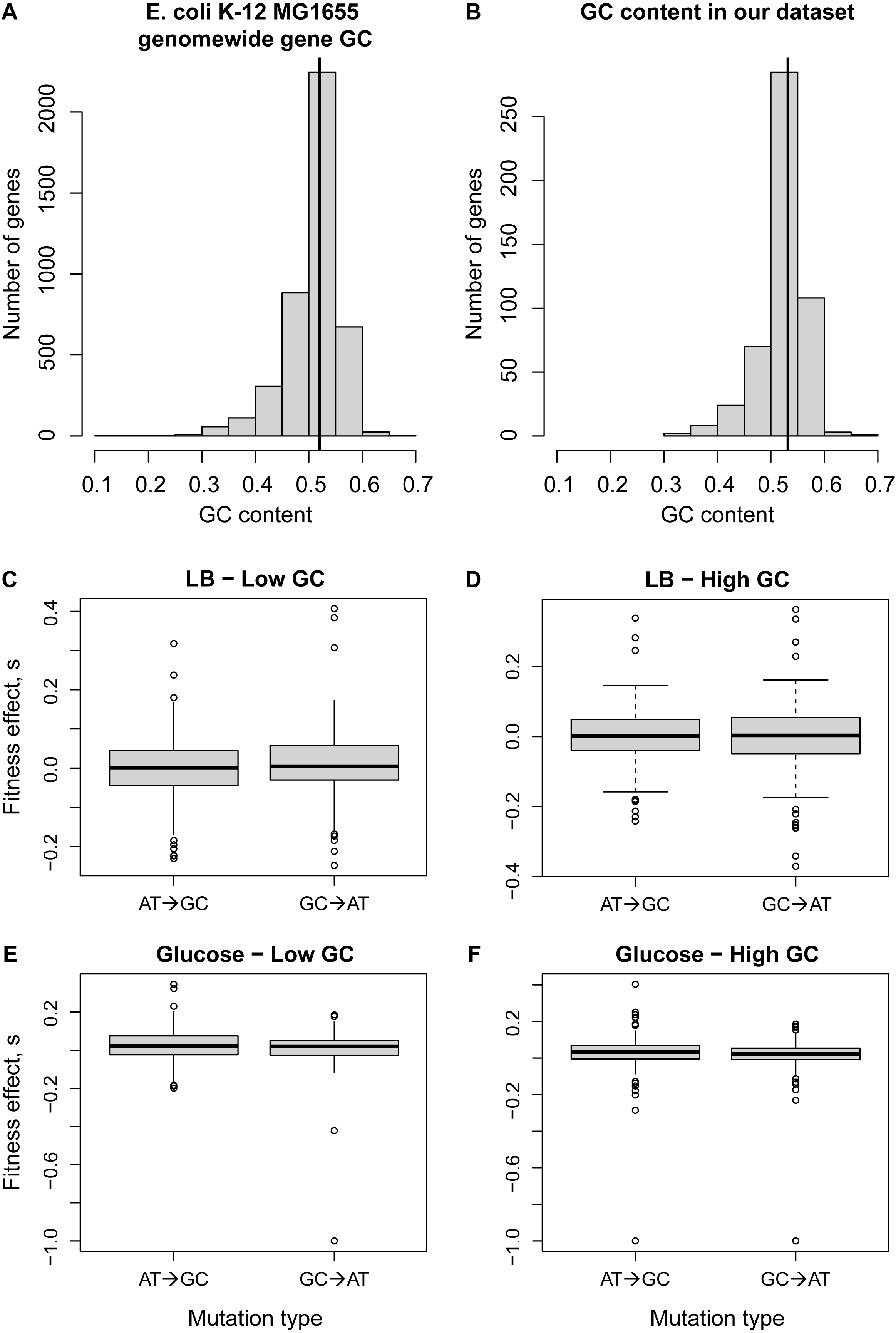

Supplement: S15 Fig — Histograms show the distribution of gene GC content in (A) E. coli K-12 MG1655 genome and (B) genes with mutations in our dataset. Vertical black lines show medians. Boxplots show fitness effects of AT→GC vs. GC→AT mutations in (C, E) low GC content genes (i.e., GC content less than the genome-wide median GC) and (D, F) high GC content genes (i.e., GC content greater than the genome-wide median GC) in (C–D) LB and (E–F) Glucose. Mutational effects were not significantly different across any of the categories shown in these plots (Wilcoxon’s rank-sum tests, p > 0.05). Data underlying this figure are given in S23 Data. (TIF) [file pbio.3003282.s015.tif]
