# Supplementary material for: Mutation bias alters the distribution of fitness effects of mutations
Source: PLoS Biol. 2025 Jul 14;23(7):e3003282. doi: 10.1371/journal.pbio.3003282 (PMC12273949; doi:10.1371/journal.pbio.3003282)
Supplement: S1 Table — (DOCX) [file pbio.3003282.s016.docx]

**S1 Table. Summary of sequencing methods used in this study, and outcomes.**

| **Strain** | **Block** | **Type** | **Lines with good sequence (of X evolved)^1^** | **Days evolved** | **Lines with one mutation** | **Library preparation method** | **Sequencing platform** | **Mean sequencing depth (range)** | **Ref** |
| --- | --- | --- | --- | --- | --- | --- | --- | --- | --- |
| ∆mutS | 1 | Anc | 1 | 0 | NA | α | A | 114x | This study |
|  |  | Evo | 295 (300) | 1 | 78 |  | Lines 1-48: A;  Lines 49-300: B | 54x  (19x - 91x) |  |
|  | 2 | Anc | 1 | 0 | NA |  | B | 43x |  |
|  |  | Evo | 50 (50) | 1 | 14 |  | B | 49x  (19x - 67x) |  |
| ∆mutL | 1 | Anc | 1 | 0 | NA | α | A | 88x | This study |
|  |  | Evo | 294 (300) | 1 | 82 |  | Lines 1-48: A;  Lines 49-192: B; Lines 193-300: A | 53x  (18x - 126x) |  |
|  | 2 | Anc | 1 | 0 | NA |  | A | 89x |  |
|  |  | Evo | 50 (50) | 1 | 15 |  | A | 80x  (50x - 132x) |  |
| ∆mutH | 1 | Anc | 1 | 0 | NA | α | A | 118x | This study |
|  |  | Evo | 297 (300) | 1 | 83 |  | Lines 1-48: A;  Lines 49-300: B | 52x  (19x - 104x) |  |
|  | 2 | Anc | 1 | 0 | NA |  | A | 66x |  |
|  |  | Evo | 49 (50) | 1 | 17 |  | A | 79x  (29x - 138x) |  |
| ∆nth-nei | 1 | Anc | 1 | 0 | NA | α | A | 89x | This study |
|  |  | Evo | 80 (80) | 8 | 33 |  | Lines 1-76: B;  Lines 77-80: A | 61x  (20x - 128x) |  |
|  | 2 | Anc | NA^a^ | NA | NA | α | NA | NA |  |
|  |  | Evo | 205 (220) | 8 | 69 |  | A | 63x  (22x - 197x) |  |
| WT | 1 | Anc | 1 | 0 | 0 | β | C | 55x | [1,2] |
|  |  | Evo | 38 (38) | 300 | 80^b^ |  | A | 103x  (19x – 220x) |  |
|  | 2 | Anc | NA^c^ | NA | NA | α | NA | NA | This study |
|  |  | Evo | 58 (60) | 85 | 14 |  | B | 64x  (42x - 88x) |  |
| ∆mutY | 1 | Anc | 1 | 0 | 0 | β | C | 37x | [2] |
|  |  | Evo | 299 (300) | 12 | 79 |  | B | 82x  (28x - 178x) |  |
|  | 2 | Anc | NA^d^ | NA | NA | β | NA | NA | This study |
|  |  | Evo | 77 (80) | 5^e^ | 26 |  | B | 42x  (21x - 61x) |  |
|  | 3^f^ | Anc | 1 | 0 | 0 |  | A | 48x |  |
|  |  | Evo | 50 (50) | 1 | 8 |  | A | 74x  (47x - 109x) |  |
| ∆mutT | 1 | Anc | 1 | 0 | 0 | α | A | 59x | This study |
|  |  | Evo | 271 (300) | 1 | 97 |  | Lines 1-128: A;  Lines 129-214: B; Lines 215-242: A; Lines 265-300: B; Lines 243-264: not sequenced | 55x  (20x - 113x) |  |

1: Lines that were not successfully sequenced (i.e., where we did not obtain sufficient good quality sequencing data or where sequencing failed entirely) are reported here, but were excluded from further analyses.

α: Illumina DNA Library preparation kit

β: Illumina Nextera XT DNA library preparation kit

A: HiSeq 2500 2x100bp PE

B: HiSeq 2500 2x125bp PE

C: MiSeq 2x250bp PE

^a, c, d^ This experimental block of MA was started from the same ancestral clone as Block 1, for the respective strain.

^b^ We sequenced 6 timepoints from each MA line, identifying 33 clones that had a single mutation and 29, 13 and 5 clones carrying 2, 3 or 4 mutations respectively, compared to the original ancestor. Given the low number of first-step mutants, we included the 2-, 3- and 4- step mutation clones in our dataset, but calculated their fitness relative to the immediate mutational ancestor (with the known mutation from the previously sequenced timepoint). We thus obtained a total of 80 single-mutational-step clones from 38 MA lines; see Sane et al., Evolution, 2018 for more details.

^e^ This block was evolved for fewer days than Block 1, because sequencing data from Block 1 led us to revise the estimated mutation rate, allowing us to shorten the expected time to a single mutation.

^f^ This block was supposed to serve as Block 2 for ∆mutT and the lines were therefore evolved for a single day. However, by mistake we used the ∆mutY ancestor to found the MA lines, so it was included in our dataset as Block 3 of ∆mutY instead of Block 2 of ∆mutT.
