# Supplementary material for: Mutation bias alters the distribution of fitness effects of mutations
Source: PLoS Biol. 2025 Jul 14;23(7):e3003282. doi: 10.1371/journal.pbio.3003282 (PMC12273949; doi:10.1371/journal.pbio.3003282)
Supplement: S2 Table — Values in bold highlight significant differences. Benjamini-Hochberg corrections for multiple comparisons were performed across all tests. (DOCX) [file pbio.3003282.s017.docx]

**S2 Table. Output of chi-square tests comparing the proportion of beneficial, neutral, and deleterious mutations across strains in LB.** Values in bold highlight significant differences. Benjamini-Hochberg corrections for multiple comparisons were performed across all tests.

| **Comparison** | **Chi-sq. statistic** | **P (Benjamini-Hochberg corrected)** |
| --- | --- | --- |
| ∆mutS – ∆mutL | 0.12 | 8.44E-01 |
| ∆mutS – ∆mutH | 0.00 | 1.00E+00 |
| ∆mutS – ∆nth-nei | 1.21 | 4.09E-01 |
| ∆mutS – WT | 1.38 | 4.09E-01 |
| ∆mutS – ∆mutY | 33.03 | **9.52E-08** |
| ∆mutS – ∆mutT | 6.59 | **2.75E-02** |
| ∆mutL – ∆mutH | 0.06 | 8.94E-01 |
| ∆mutL – ∆nth-nei | 0.20 | 8.05E-01 |
| ∆mutL – WT | 0.29 | 7.73E-01 |
| ∆mutL – ∆mutY | 29.74 | **3.46E-07** |
| ∆mutL – ∆mutT | 4.13 | **4.20E-02** |
| ∆mutH – ∆nth-nei | 1.03 | 4.33E-01 |
| ∆mutH – WT | 1.20 | 4.09E-01 |
| ∆mutH – ∆mutY | 34.79 | **7.73E-08** |
| ∆mutH – ∆mutT | 6.55 | **2.75E-02** |
| ∆nth-nei – WT | 0.00 | 1.00E+00 |
| ∆nth-nei – ∆mutY | 25.14 | **2.79E-06** |
| ∆nth-nei – ∆mutT | 1.92 | 3.46E-01 |
| WT – ∆mutY | 22.82 | **7.48E-06** |
| WT – ∆mutT | 1.55 | **4.06E-01** |
| ∆mutY – ∆mutT | 12.49 | **1.43E-03** |
