# Supplementary material for: Mutation bias alters the distribution of fitness effects of mutations
Source: PLoS Biol. 2025 Jul 14;23(7):e3003282. doi: 10.1371/journal.pbio.3003282 (PMC12273949; doi:10.1371/journal.pbio.3003282)
Supplement: S3 Table — Values in bold highlight significant differences. Benjamini–Hochberg corrections for multiple comparisons were performed across all tests. (DOCX) [file pbio.3003282.s018.docx]

**S3 Table. Output of chi-square tests comparing the proportion of beneficial, neutral, and deleterious mutations across strains in glucose.** Values in bold highlight significant differences. Benjamini-Hochberg corrections for multiple comparisons were performed across all tests.

| **Comparison** | **Chi-sq. statistic** | **P (Benjamini-Hochberg corrected)** |
| --- | --- | --- |
| ∆mutS – ∆mutL | 7.19 | **1.28E-02** |
| ∆mutS – ∆mutH | 8.22 | **7.9E-03** |
| ∆mutS – ∆nth-nei | 2.96 | 1.28E-01 |
| ∆mutS – WT | 5.82 | **2.55E-02** |
| ∆mutS – ∆mutY | 33.28 | **8.35E-08** |
| ∆mutS – ∆mutT | 34.93 | **7.61E-08** |
| ∆mutL – ∆mutH | 17.48 | **3.74E-04** |
| ∆mutL – ∆nth-nei | 1.55 | 4.85E-01 |
| ∆mutL – WT | 3.61 | 1.86E-01 |
| ∆mutL – ∆mutY | 10.37 | **8.41E-03** |
| ∆mutL – ∆mutT | 13.09 | **2.75E-03** |
| ∆mutH – ∆nth-nei | 26.15 | **8.80E-06** |
| ∆mutH – WT | 5.85 | 6.63E-02 |
| ∆mutH – ∆mutY | 26.95 | **7.38E-06** |
| ∆mutH – ∆mutT | 30.76 | **1.47E-06** |
| ∆nth-nei – WT | 7.57 | **2.98E-02** |
| ∆nth-nei – ∆mutY | 18.15 | **3.00E-04** |
| ∆nth-nei – ∆mutT | 20.29 | **1.24E-04** |
| WT – ∆mutY | 16.31 | **6.02E-04** |
| WT – ∆mutT | 20.18 | **1.24E-04** |
| ∆mutY – ∆mutT | 0.66 | 7.19E-01 |
