# Supplementary material for: Mutation bias alters the distribution of fitness effects of mutations
Source: PLoS Biol. 2025 Jul 14;23(7):e3003282. doi: 10.1371/journal.pbio.3003282 (PMC12273949; doi:10.1371/journal.pbio.3003282)
Supplement: S4 Table — Supply of beneficial mutations (Sb) is calculated for each strain in both environments (LB and Glucose) using the empirically estimated fb values (Fig 4A), whole-genome mutation rates (µ, Table 1), and genome size (4,641,652 bp) as: Sb = fb × µ × genome size. Beneficial supply assuming a WT DFE, Sb(WT DFE), is calculated as fb(WT) × µ × genome size. Sb and Sb(WT DFE) relative to WT are reported in the two rightmost columns. Confidence intervals were calculated as 1.96 × (standard deviation of Sb). (DOCX) [file pbio.3003282.s019.docx]

**S4 Table. Beneficial supply calculations for all strains.** Supply of beneficial mutations (S_b_) is calculated for each strain in both environments (LB and Glucose) using the empirically estimated f_b_ values (Figure 4A), whole genome mutation rates (µ, Table 1), and genome size (4641652 bp) as: S_b_ = f_b_ x µ x genome size. Beneficial supply assuming a WT DFE, S_b(WT DFE)_, is calculated as f_b(WT)_ x µ x genome size. S_b_ and S_b(WT DFE)_ relative to WT are reported in the two rightmost columns. Confidence intervals were calculated as 1.96 x (standard deviation of S_b_).

| **Strain** | **Env** | **f_b_** | **µ** | **S_b_** | **S_b(WT DFE)_** | **S_b_/S_b(WT)_** | **S_b(WT DFE)_/**  **S_b(WT)_** |
| --- | --- | --- | --- | --- | --- | --- | --- |
| ∆mutS | LB | 0.032 | 1.44E-08 | 2.14E-03 | 5.54E-03 | 60.35 | 156.53 |
|  |  | ±0.04 |  | ±2.27E-03 |  |  |  |
| ∆mutL | LB | 0.053 | 1.41E-08 | 3.47E-03 | 5.44E-03 | 98.12 | 153.67 |
|  |  | ±0.04 |  | ±2.62E-03 |  |  |  |
| ∆mutH | LB | 0.035 | 2.11E-08 | 3.43E-03 | 8.14E-03 | 97.01 | 230.06 |
|  |  | ±0.04 |  | ±2.23E-03 |  |  |  |
| ∆nth_nei | LB | 0.079 | 1.77E-09 | 6.50E-04 | 6.83E-04 | 18.37 | 19.30 |
|  |  | ±0.05 |  | ±3.06E-04 |  |  |  |
| WT | LB | 0.083 | 9.18E-11 | 3.54E-05 | 3.54E-05 | 1.00 | 1.00 |
|  |  | ±0.06 |  | ±3.70E-05 |  |  |  |
| ∆mutY | LB | 0.379 | 8.34E-10 | 1.47E-03 | 3.21E-04 | 41.48 | 9.08 |
|  |  | ±0.09 |  | ±7.06E-04 |  |  |  |
| ∆mutT | LB | 0.151 | 2.34E-08 | 1.64E-02 | 9.00E-03 | 462.63 | 254.29 |
|  |  | ±0.07 |  | ±5.47E-03 |  |  |  |
| ∆mutS | Glu | 0.242 | 1.44E-08 | 1.61E-02 | 2.80E-02 | 90.19 | 156.53 |
|  |  | ±0.09 |  | ±5.52E-03 |  |  |  |
| ∆mutL | Glu | 0.393 | 1.41E-08 | 2.57E-02 | 2.75E-02 | 143.79 | 153.67 |
|  |  | ±0.10 |  | ±5.71E-03 |  |  |  |
| ∆mutH | Glu | 0.451 | 2.11E-08 | 4.42E-02 | 4.12E-02 | 247.04 | 230.06 |
|  |  | ±0.10 |  | ±6.04E-03 |  |  |  |
| ∆nth_nei | Glu | 0.367 | 1.77E-09 | 3.02E-03 | 3.46E-03 | 16.87 | 19.30 |
|  |  | ±0.09 |  | ±5.46E-04 |  |  |  |
| WT | Glu | 0.42 | 9.18E-11 | 1.79E-04 | 1.79E-04 | 1.00 | 1.00 |
|  |  | ±0.10 |  | ±6.62E-05 |  |  |  |
| ∆mutY | Glu | 0.658 | 8.34E-10 | 2.55E-03 | 1.63E-03 | 14.23 | 9.08 |
|  |  | ±0.09 |  | ±6.91E-04 |  |  |  |
| ∆mutT | Glu | 0.683 | 2.34E-08 | 7.40E-02 | 4.55E-02 | 413.53 | 254.29 |
|  |  | ±0.09 |  | ±7.10E-03 |  |  |  |
