# Supplementary material for: Mutation bias alters the distribution of fitness effects of mutations
Source: PLoS Biol. 2025 Jul 14;23(7):e3003282. doi: 10.1371/journal.pbio.3003282 (PMC12273949; doi:10.1371/journal.pbio.3003282)
Supplement: S5 Table — Deleterious load (Ld) is calculated for each strain in both environments (LB and Glucose) using the empirically estimated fd values (Fig 4A), whole-genome mutation rates (µ) (Table 1), and genome size (4,641,652 bp) as: Ld = fd × µ × genome size. Deleterious load assuming a WT DFE, Ld(WT DFE), is calculated as fd(WT) × µ × genome size. Ld and Ld(WT DFE) relative to WT are reported in the two rightmost columns. Confidence intervals were calculated as 1.96 × (standard deviation of Ld). (DOCX) [file pbio.3003282.s020.docx]

**S5 Table. Deleterious load calculations for all strains.** Deleterious load (L_d_) is calculated for each strain in both environments (LB and Glucose) using the empirically estimated f_d_ values (Figure 4A), whole genome mutation rates (µ) (Table 1), and genome size (4641652 bp) as: L_d_ = f_d_ x µ x genome size. Deleterious load assuming a WT DFE, L_d(WT DFE)_, is calculated as f_d(WT)_ x µ x genome size. L_d_ and L_d(WT DFE)_ relative to WT are reported in the two rightmost columns. Confidence intervals were calculated as 1.96 x (standard deviation of L_d_).

| **Strain** | **Env** | **f_d_** | **µ** | **L_d_** | **L_d(WT DFE)_** | **L_d_ /L_d(WT)_** | **L_d(WT DFE)_/**  **L_d(WT)_** | |
| --- | --- | --- | --- | --- | --- | --- | --- | --- |
| ∆mutS | LB | 0.44 | 1.44E-08 | 2.91E-02 | 3.94E-02 | 115.67 | | 156.53 |
|  |  | ±0.10 |  | ±6.39E-03 |  |  | |  |
| ∆mutL | LB | 0.43 | 1.41E-08 | 2.82E-02 | 3.87E-02 | 111.99 | | 153.67 |
|  |  | ±0.10 |  | ±5.79E-03 |  |  | |  |
| ∆mutH | LB | 0.71 | 2.11E-08 | 7.00E-02 | 5.79E-02 | 278.41 | | 230.06 |
|  |  | ±0.09 |  | ±5.48E-03 |  |  | |  |
| ∆nth-nei | LB | 0.22 | 1.77E-09 | 1.83E-03 | 4.85E-03 | 7.26 | | 19.30 |
|  |  | ±0.08 |  | ±4.71E-04 |  |  | |  |
| WT | LB | 0.59 | 9.18E-11 | 2.52E-04 | 2.52E-04 | 1.00 | | 1.00 |
|  |  | ±0.10 |  | ±6.60E-05 |  |  | |  |
| ∆mutY | LB | 0.29 | 8.34E-10 | 1.12E-03 | 2.28E-03 | 4.46 | | 9.08 |
|  |  | ±0.08 |  | ±6.61E-04 |  |  | |  |
| ∆mutT | LB | 0.13 | 2.34E-08 | 1.36E-02 | 6.40E-02 | 53.88 | | 254.29 |
|  |  | ±0.07 |  | ±5.05E-03 |  |  | |  |
| ∆mutS | Glu | 0.45 | 1.44E-08 | 2.99E-02 | 2.03E-02 | 230.67 | | 156.53 |
|  |  | ±0.10 |  | ±6.41E-03 |  |  | |  |
| ∆mutL | Glu | 0.23 | 1.41E-08 | 1.51E-02 | 1.99E-02 | 116.77 | | 153.67 |
|  |  | ±0.08 |  | ±4.93E-03 |  |  | |  |
| ∆mutH | Glu | 0.41 | 2.11E-08 | 4.00E-02 | 2.98E-02 | 308.76 | | 230.06 |
|  |  | ±0.10 |  | ±5.96E-03 |  |  | |  |
| ∆nth-nei | Glu | 0.18 | 1.77E-09 | 1.48E-03 | 2.50E-03 | 11.43 | | 19.30 |
|  |  | ±0.07 |  | ±4.35E-04 |  |  | |  |
| WT | Glu | 0.30 | 9.18E-11 | 1.30E-04 | 1.30E-04 | 1.00 | | 1.00 |
|  |  | ±0.09 |  | ±6.17E-05 |  |  | |  |
| ∆mutY | Glu | 0.10 | 8.34E-10 | 3.95E-04 | 1.18E-03 | 3.05 | | 9.08 |
|  |  | ±0.06 |  | ±4.41E-04 |  |  | |  |
| ∆mutT | Glu | 0.07 | 2.34E-08 | 7.59E-03 | 3.30E-02 | 58.55 | | 254.29 |
|  |  | ±0.05 |  | ±3.90E-03 |  |  | |  |
