# Supplementary material for: Mutation bias alters the distribution of fitness effects of mutations
Source: PLoS Biol. 2025 Jul 14;23(7):e3003282. doi: 10.1371/journal.pbio.3003282 (PMC12273949; doi:10.1371/journal.pbio.3003282)
Supplement: S6 Table — The table shows differences between fitness effects of different types of mutations between pairs of strains. Values in bold highlight significant differences. (DOCX) [file pbio.3003282.s021.docx]

**S6 Table. Fitness effects of mutations associated with other axes of mutation bias.** The table shows differences between fitness effects of different types of mutations between pairs of strains. Values in bold highlight significant differences.

| **Mutation type** | **Comparison** | **P (Benjamini-Hochberg corrected)** | |
| --- | --- | --- | --- |
|  |  | **LB** | **Glucose** |
| coding | ∆mutL – ∆mutH | 0.538 | **0.002** |
| coding | ∆mutS – ∆mutH | 0.297 | 0.134 |
| coding | ∆mutS – ∆mutL | 0.539 | **0.001** |
| coding | ∆nth-nei – ∆mutH | 0.063 | 0.103 |
| coding | ∆nth-nei – ∆mutL | 0.063 | 0.050 |
| coding | ∆nth-nei – ∆mutS | 0.209 | **0.014** |
| coding | WT – ∆mutH | **0.009** | 0.346 |
| coding | WT – ∆mutL | 0.063 | **0.014** |
| coding | WT – ∆mutS | 0.098 | 0.106 |
| coding | WT – ∆nth-nei | 0.297 | 0.346 |
| noncoding | ∆mutL – ∆mutH | 0.989 | **0.018** |
| noncoding | ∆mutS – ∆mutH | 0.795 | 0.572 |
| noncoding | ∆mutS – ∆mutL | 0.613 | 0.109 |
| noncoding | ∆nth-nei – ∆mutH | 0.795 | 0.064 |
| noncoding | ∆nth-nei – ∆mutL | 0.334 | 0.352 |
| noncoding | ∆nth-nei – ∆mutS | **0.018** | 0.572 |
| noncoding | WT – ∆mutH | 0.519 | 0.791 |
| noncoding | WT – ∆mutL | 0.519 | **0.045** |
| noncoding | WT – ∆mutS | 0.519 | 0.747 |
| noncoding | WT – ∆nth-nei | 0.989 | 0.109 |
| syn | ∆mutL – ∆mutH | 0.897 | 0.415 |
| syn | ∆mutS – ∆mutH | 0.897 | 0.534 |
| syn | ∆mutS – ∆mutL | 0.897 | 0.415 |
| syn | ∆nth-nei – ∆mutH | 0.135 | 0.671 |
| syn | ∆nth-nei – ∆mutL | 0.135 | 0.534 |
| syn | ∆nth-nei – ∆mutS | 0.266 | 0.415 |
| syn | WT – ∆mutH | 0.093 | 0.691 |
| syn | WT – ∆mutL | 0.104 | 0.415 |
| syn | WT – ∆mutS | 0.104 | 0.453 |
| syn | WT – ∆nth-nei | 0.583 | 0.560 |
| nonsyn | ∆mutL – ∆mutH | 0.552 | **0.006** |
| nonsyn | ∆mutS – ∆mutH | 0.552 | 0.256 |
| nonsyn | ∆mutS – ∆mutL | 0.552 | **0.006** |
| nonsyn | ∆nth-nei – ∆mutH | 0.199 | 0.118 |
| nonsyn | ∆nth-nei – ∆mutL | 0.199 | 0.118 |
| nonsyn | ∆nth-nei – ∆mutS | 0.552 | 0.076 |
| nonsyn | WT – ∆mutH | 0.199 | 0.181 |
| nonsyn | WT – ∆mutL | 0.552 | 0.147 |
| nonsyn | WT – ∆mutS | 0.653 | 0.118 |
| nonsyn | WT – ∆nth-nei | 0.997 | 0.838 |
| AT🡪GC | ∆mutL – ∆mutH | 0.973 | **0.000** |
| AT🡪GC | ∆mutS – ∆mutH | 0.973 | 0.695 |
| AT🡪GC | ∆mutS – ∆mutL | 0.973 | **0.002** |
| AT🡪GC | ∆nth-nei – ∆mutH | 0.973 | 0.695 |
| AT🡪GC | ∆nth-nei – ∆mutL | 0.973 | 0.292 |
| AT🡪GC | ∆nth-nei – ∆mutS | 0.973 | 0.695 |
| AT🡪GC | WT – ∆mutH | 0.149 | 0.342 |
| AT🡪GC | WT – ∆mutL | 0.167 | 0.181 |
| AT🡪GC | WT – ∆mutS | 0.149 | 0.342 |
| AT🡪GC | WT – ∆nth-nei | 0.903 | 0.712 |
| GC🡪AT | ∆mutL – ∆mutH | 0.667 | 0.256 |
| GC🡪AT | ∆mutS – ∆mutH | 0.317 | 0.256 |
| GC🡪AT | ∆mutS – ∆mutL | 0.685 | **0.029** |
| GC🡪AT | ∆nth-nei – ∆mutH | 0.317 | 0.577 |
| GC🡪AT | ∆nth-nei – ∆mutL | 0.667 | 0.512 |
| GC🡪AT | ∆nth-nei – ∆mutS | 0.880 | **0.029** |
| GC🡪AT | WT – ∆mutH | 0.317 | 0.828 |
| GC🡪AT | WT – ∆mutL | 0.812 | 0.256 |
| GC🡪AT | WT – ∆mutS | 0.880 | 0.202 |
| GC🡪AT | WT – ∆nth-nei | 0.880 | 0.256 |
