# Supplementary material for: Mutation bias alters the distribution of fitness effects of mutations
Source: PLoS Biol. 2025 Jul 14;23(7):e3003282. doi: 10.1371/journal.pbio.3003282 (PMC12273949; doi:10.1371/journal.pbio.3003282)
Supplement: Attachment — Submitted filename: ResponseToReviewers.pdf [file pbio.3003282.s052.pdf]

We thank the reviewers for their detailed and constructive comments, which have been very helpful in improving our manuscript. **Briefly, we have made the following major changes**:

1. To clarify the logic leading to the main prediction being tested in this study, we modified the introduction and abstract and added a new schematic
2. We conducted new analyses to verify single-mutation clones, including estimates of false positive and false negative rates
3. We conducted new analyses to test the effects of more conservative mutation calling on DFEs
4. We have added detailed information on sequencing and mutation calling

Please find below our point-by-point responses to all reviewer comments in blue. We have also provided a manuscript file with marked changes to aid review.

Reviewer #1

MS Summary: This paper examines how varying mutation biases in Escherichia coli can influence the distribution of fitness effects (DFE), potentially shaping adaptive outcomes. Utilising mutator strains to manipulate mutation biases in E. coli, this study offers empirical data to a theoretical prediction that reversing a mutation bias should shift the DFE towards more advantageous mutations, thus facilitating adaptation. Results robustly support the hypothesis: strains exhibiting a transversion bias, which reverses the usual transition bias observed in wild-type E. coli, display a DFE characterised by a higher proportion of beneficial mutations. In contrast, strains that maintain or enhance the native transition bias demonstrate a reduced frequency of advantageous mutations.

Major comments: None - This paper addresses an interesting and broadly relevant question in evolutionary biology, the role of mutation biases in driving adaptive outcomes. This paper uses an elegant experimental design to provide empirical support to a largely theoretical area. I have no problems with the experimental design, which is appropriate for the hypothesis being tested. I have a few minor queries which are generally related to providing additional information for clarification.

Thank you for your encouraging comments.

Minor comments:

1. In the introduction, when discussing whether the DFE should shift left or right under changes in mutation bias, I think it would be helpful to have a conceptual figure, that allows readers unfamiliar with DFEs to understand what the axes (and therefore shifts) represent in terms of population fitness.

We have now added a schematic (Figure 1) to illustrate DFE shifts and highlight the key prediction tested in this study.

2. Page 13, line 6: where the authors write, "measured the fitness effect of each mutation in two environments", I think it would be useful to reference each of the environments here.

We have now done this.

3. Table 1: I understand that the details of the mechanism for each mutator strain is given as a footnote in Table 1, but I feel this information could be made more obvious to the reader. Either as a separate table or a conceptual figure.

We have added additional text mentioning some of this information in the introduction (paragraph 4) so that readers do not miss seeing it in Table 1.

4. Page 14, line 11: What was the rationale for the number of founder lines in each mutator treatment group?

Our goal was to obtain ~100 clones with single mutations for each strain. Given the 10 to 100-fold difference in mutation rates, we could either do a short-term MA experiment (for strains with high mutation rate) or a longer-term MA experiment (for strains with low mutation rate). Logistically it was easier to do many lineages of the former, and fewer lineages of the latter. Hence, the number of founder lines for each strain depended on the feasibility and length of the MA experiment. We have now described this in detail in the Materials and Methods section (subsection “mutation accumulation experiments”, paragraph 2).

5. Page 14, line 18: I assume by "set" the authors mean experimental block, but perhaps this could be clarified in the text. In addition, there should be justification for why different "sets" were used across the different treatments and whether this was factored into the statistical analysis. Finally, it wasn't clear to me why ΔmutY was the only treatment where sets that were run for different lengths of time were used. Could any additional insights be made from this?

We now say “experimental block” throughout for clarity. Different numbers of blocks were used across treatments for two main reasons: 1. To increase the number of lines with single mutations after a set was performed and sequenced, and 2. For logistical reasons (handling too many MA lines at once is difficult). We have now explained this reasoning briefly in the Methods section (subsection “mutation accumulation experiments”, paragraph 2).

For ΔmutY, we used the larger set of MA lines (block 1, 300 lines) to obtain a more accurate estimate of the mutation rate, and used this to calculate the number of generations required to accumulate 1 mutation. We used this to determine the length of the experiment for the block 2 lines. Block 3 was an inadvertent error: these were meant to be ΔmutT MA lines, to be evolved for 1 day given the very high mutation rate. However, instead of ΔmutT we erroneously set up 50 ΔmutY MA lines, which were stopped after 1 day as planned (for ΔmutT). We discovered the error too late (after WGS), but since we found some lines with a single mutation each, we included them as an additional MA block for ΔmutY. We have now explained these oddities in the footnotes to Table S1.

6. Page 14, Line 20: Can the authors provide the estimated mutation rates for each of the lines (perhaps included in a table/figure as suggested in a point above)? It would be useful if the authors could provide the expected average number of mutations across lines for each strain over the course of the experiment.

We have now added this information in Supplementary datasheet S2.

7. Page 15, Line 19: When analysing genomes for mutations, were the default parameters used for Breseq and VarScan?

Yes; this is now mentioned in the Methods (subsection “whole genome sequencing…”, paragraph 2).

8. Page 15, Line 28: How did the authors calculate the number of generations for each strain?

We had previously estimated the number of generations the WT strain goes through in 24 hours on LB agar plates (Sane et al., Evolution, 2018). Assuming that our mutator strains do not differ from the WT in growth on LB agar, we used this estimate to calculate the number of generations elapsed in our MA experiments. We have now added this information in the Methods (subsection “mutation accumulation experiments”, paragraph 3).

9. General point and possibly personal preference (but will also avoid misinterpretation), where formulas are given in the text, can they be represented as equations rather than embedded in the text to avoid confusion, as / can be either/or rather than divide.

We have now fixed this as suggested.

10. Figure 1: The schematic in the right bottom corner of Figure 1 and 2 is the same. Would it be best to show this only once? As presented, it suggests it might differ between environments.

We think it is helpful if the schematic is included in both figures. We have now clarified in the legend to Figure 2 (now Figure 3) that the schematic is identical to that in Figure 1 (now Figure 2).

11. Figure 3: It is not stated in the figure legend that the different colours correspond to the different biases.

We have now added this information.

Reviewer #2

Sane, et al. use repair mutants to manipulate the mutation spectrum in E. coli, then use paired growth experiments to interrogate the DFE for the mutant strains. They argue that reversing the previous ti/tv bias shifts the DFE to the right, while enhancing the previous bias shifts it to the left. This kind of result suggests that conditions in nature are conducive for a role of bias-shifts.

I really enjoyed reading about this project. From the experimental set-up, my initial concern was how to deal with the possibility that the different DFEs reflect something other than just the ti/tv bias. But the authors handled this well. This is one of the strengths of the paper.

Thank you for your encouraging comments.

However, I have 2 other main concerns and one minor concern.

statistical hypothesis-testing

The first main concern is that the statistical approach does not take into account the structure of the data and the history of how this experiment emerged.

The mutation spectrum is the independent variable in this experiment (and the DFE is the dependent variable), but it is difficult to modify— it has to be done via repair mutants that often have idiosyncratic effects via repair pathways. MutY and mutT both affect the 8-oxo-GTP damage-repair pathway and have similar spectra. MutHLS affect the mismatch repair pathway and have similar spectra. The nth-nei deletion affects a third pathway. So, rather than wild-type plus 6 independent mutant versions of the mutation spectrum, we have something more like wild-type plus 3 mutant types: MMR-deficient (3 versions), 8-oxo-GTP-deficient (2 versions), and nth-nei (1 version).

Note that the authors group the strains this way in Table 1 with the notes designated with dagger, psi and phi. So, they aren't really hiding this, but they also aren't taking it into account in the data analysis.

If we look at the regressions in Fig. S3 in this way, with 4 points instead of 7, the results are no longer clear. In every case, if we take out the 8-oxo-GTP-deficient spectrum, there is no correlation. This is very important because, in fact, the authors reported previously in Sane, et al. 2023 that a mutY strain with higher transversion bias had a right-shifted DFE. As a matter of statistical hygiene, we would be justified in removing the data from 8-oxo-GTP-deficient mutants on the grounds that it represents prior knowledge, not independent relative to the hypothesis to be tested, which was inspired by these earlier results.

I have attached a modified version of Fig. S3 to illustrate this.

Fig S3 and the accompanying statistical tests really should be front and center in the presentation. This is the figure that gives the most direct and accurate depiction of the test that the authors wish to perform.

So, a minimal revision would be to bring S3 into the main text and re-do the statistics. I suspect that

this will seriously weaken the authors' conclusions.

We see the point being raised here, but we do not agree that the previously known data points (WT and mutY) should be removed from the current analysis, for the following reasons. First, at the end of our prior study we proposed a general hypothesis, of which only one aspect was addressed in that paper. Here, we test the full hypothesis using several additional strains, and the comparison of each strain with the WT is a critical aspect (please see below for a more detailed discussion). Second, in this study we added more mutants to the WT and mutY datasets, increasing the precision with which we determine the DFEs; and the measurements we report here were conducted in a slightly different experimental setup (96 well rather than 48 well plates).

We emphasize that in this study we focus on testing the hypothesis that bias reversal will cause right-shifted DFEs but bias reinforcement will cause left-shifted DFEs. This requires us to compare Ts-biased vs. Tv-biased strains to each other and to the WT. This is different from the (related, but distinct) hypothesis that a higher magnitude of bias reversal should cause increasingly larger right-shifts. Testing the latter prediction would require measuring the DFEs of many strains across a wide and (ideally) evenly distributed range of Tv bias. However, it is difficult to dial the Tv bias up or down in a continuous fashion, and to measure the DFEs of dozens of strains. Hence, this prediction was not the focus of our study. The correlation between fb and Tv bias (now Figure S11) has clear limitations (poor spread of points along the x-axis and low n) and is thus presented as an opportunistic aside, with only a brief mention in the main text. We have now clarified the distinction between the two predictions (Introduction, paragraph 3), and we clearly mention the current limitations in testing for a correlation (Results subsection “transversion-biased strains have a right-shifted…”, paragraph 2).

Grouping by repair pathway is not meaningful in our case, because though several genes act within the same pathway, they perform distinct molecular functions and influence the spectrum in different ways. This is, e.g., clear when comparing the ΔmutT and ΔmutY strains (Figure 4G). Further, as noted in the discussion section, deleting genes in the same pathway (e.g., ΔmutS, ΔmutH, ΔmutL) leads to distinct DFEs (Figure 4A). Hence, each mutator is effectively an independent data point for our analysis, driving either a bias reversal or reinforcement with respect to the WT strain.

quantized fitness values?

My second concern is that the fitness data are discretized or quantized in some way that suggests an artefact and which needs to be explained. If we look at the tick marks on the X axis in Fig. 2, we see that they are all rounded versions of n/80, e.g., 17/80 = 0.2125 and rounds to 0.213.

```
scale <- c(-0.413, -0.288, -0.163, -0.038, 0.088, 0.213, 0.338)
scale * 80
[1] -33.04 -23.04 -13.04  -3.04   7.04  17.04  27.04
```

That is not really a concern because these are arbitrary tick marks on a histogram. However, the same thing is true of the reported median fitness values:

```
vals <- c(-0.038, -0.038, -0.163, -0.013, -0.063, 0.038, 0.013)
vals * 80
[1]  -3.04  -3.04 -13.04  -1.04  -5.04   3.04   1.04
```

For instance, -5/80 = -0.0625 which rounds to -0.063.

According to the methods, the fitness values in the DFE (from which the median is drawn) are based on averages of 3 growth rates converted to a relative growth rate (by dividing by wild-type growth). Even if the growth measurements are quantized in some crude way, an average of 3 things isn't going to turn into whole fractions of 80. Note that, whereas the DFEs are corrected for ascertainment bias, this only adjusts the frequencies and not the fitness values (this is clear in S1 and S2), so it cannot be the source of quantization.

I hope that this has a trivial explanation and that the authors will find it and make any necessary

corrections or explanations.

Thank you for pointing this out. The discretization does arise due to the correction of DFEs for selection bias; because the medians were derived from corrected DFEs, they in turn have slightly odd values. As you rightly say, the fitness values themselves do not change when we correct for selection bias, but the frequency distribution (DFE) does change. The correction involves binning the measured selection coefficients and multiplying the mean value of each bin by a factor that accounts for the probability of selection favouring (or disfavouring) mutations with selection coefficients with the mean bin value. As a result, the actual fitness values in each bin are "lost", leading to the odd-looking DFE bins (and medians calculated from the binned, corrected DFEs). The factor of 80 arises because we set bin widths using our previous WT DFE that had 80 data points. We have now described the bias correction in greater detail in the Methods (subsection "growth rate measurements..", paragraph 3).

Unclear logic in regard to theory and prediction

Finally, I'm concerned that the presentation garbles the logic underlying the theory and the experiment. This begins in the abstract with the confusing and unnecessary "constraints" language and confusing references to a prediction.

There is no need to talk about imaginary "constraints" or "limits", e.g., in the latest 2024 piece from Tuffaha, et al they say "In asexual microbes, bias reversals in the mutation spectrum can speed adaptation by increasing access to previously undersampled beneficial mutations."

The authors keep talking about a theory and a prediction but they are not distinguishing biased depletion of the DBFE from evolutionary bias shifts. They haven't shown evolutionary bias shifts. They have (arguably) shown that bias shifts are plausible because biased depletion of the DBFE is (from their experiments) plausibly large enough.

You might think that these are all one theory but theoreticians in particular are going to be interested in biased depletion of the DBFE even if adaptive bias-shifts never happen in evolution, because biased depletion of the DBFE has consequences for the shape of the DBFE and for the rate of adaptation, and those are issues of concern in pop-gen.

If your theory is that adaptive bias-shifts are actually important in evolution, then it is a prerequisite of this theory that adaptive walks in nature happen in such a way that the DBFE is depleted in a biased way, and that this depletion is strong enough to make a bias-shift (driven by lineage selection) plausible. Per Tuffaha, et al, 2023 this is subject to a Goldilocks condition because, if the adaptive walk hasn't gone on long enough, there will not be enough biased depletion to to make a bias shift sufficiently beneficial, and if it has gone on too long, there won't be enough high-benefit mutations in the DBFE to matter. So, it is not a given that bias-shifts are plausible. The preconditions might be quite narrow.

BTW, the way to explain this theory to theoreticians is that the DBFE is finite and adaptive walks are subject to kinetic bias in two ways. Orr and others have shown that the larger steps in fitness have an advantage, and so they tend to take place earlier in an adaptive walk. Likewise, Stoltzfus and others have shown that the mutationally favored steps tend to occur earlier. If the DBFE is finite, it can be depleted, and because of these kinetic biases, it will be depleted in a biased way: the bigger and more mutationally likely steps will be over-sampled, and the smaller and less mutationally likely steps will be under-sampled. Those are the first-order consequences of kinetic bias for the DBFE. But there is a possible second-order effect, which is that a shift in mutation bias could be favored by lineage selection, due to an increased rate of adaptation.

Having said all of that, here is the issue: what theory, specifically, predicts a substantially biased depletion of the DBFE in your E. coli strains? On the one hand, the pop-gen principles DO NOT predict this without other assumptions, e.g., about the finiteness of the DBFE and how long adaptation has been happening under the current spectrum. On the other hand, if your theory is that bias reversals are a general feature of evolution, then this implies that there exist broad conditions under which we would find a strongly biased depletion of the DBFE. If that is your theory, then yes there is a

sense in which we anticipate that it should be easy to find evidence for strongly biased depletion of the DBFE. I want to see the logic of it laid out clearly. And if that is the theory, the discussion should cite work like Steenwyk, et al. on changes in repair pathways in evolution, and advance the interpretation that these observed changes in deep evolution are indeed adaptive bias shifts and that more evidence for shifts will be found.

Thank you for these comments.

Your point about unclear logic is well taken. We have now modified the beginning of the abstract and the Introduction (paragraph 3), to more clearly convey prior work leading to our core prediction that depletion of a finite DBFE — specifically depletion of beneficial mutations of the type favoured by an existing bias — should increase the beneficial fraction of the DFE. If true, this implies that bias reversals should be selectively favoured. However, in the current study we did not intend to test if bias reversals are a general feature of evolution, and hence we do not discuss this in detail. But these are important questions that we now highlight in the last paragraph of the Discussion. We have also made sure that we clearly distinguish between the direct expectation/observation of higher fb values in bias-reversed strains vs. the implication of a selective advantage of reversals.

It is true that there is no specific reason to expect that *E. coli* (or any given species) should have a DBFE that conforms to the necessary conditions. Importantly, Tuffaha et al show that the conditions favouring a bias reversal are quite broad, e.g., including a modest depletion of the DBFE, clonal interference, and varying degrees of epistasis, suggesting that the Goldilocks zone may not be prohibitively narrow. Still, we cannot be certain that the conditions will be met for a given population. However, it seems likely that, given widespread pleiotropic effects of mutations and multiple and changing selection pressures, any organism will never be close to achieving the maximum possible f_b (beneficial fraction of mutations) value of 100%. Hence, we might speculate that any population that has evolved in any environment will always have a “depleted” DBFE; indeed, most empirical studies indicate that there are many more deleterious than beneficial mutations. Still, we don’t know exactly how depleted DBFEs are, and how depleted they need to be for a bias reversal to be advantageous in a particular species given other relevant factors such as mutation rate, bias, population size, epistasis, etc. Our approach was to do the work and experimentally estimate the DFE in different environments, hoping that the DBFE might be appropriately depleted in some of them. In our prior work (Sane et al 2023) we stumbled upon an instance when the necessary conditions for advantageous bias reversals were apparently met, in a mutY strain of *E coli.* In the current work, we tested for generality, by measuring the DFE of one more bias-reversed strain, and importantly, of several bias-reinforcing strains, in both rich and poor growth media.

Should this also work in most other species? As noted above, prior theoretical work and simulations indicate that the answer might be “yes”. However, for the reasons outlined above, it is not easy to answer this question empirically. We did try to do this earlier (Sane et al 2023), using phylogenetic analyses to show that DNA repair gene gains/losses were consistent with frequent bias reversals in bacterial lineages. In that paper, we had also discussed other work demonstrating frequent changes in DNA repair genes and pathways in several taxa, the limitations of such analyses for understanding bias shifts, and the need for further work. We have now briefly highlighted these questions in the Discussion (last paragraph).

Some other small comments:

* abstract says "hundreds of single muts...for each strain" but it is only ~100 for each strain

This is now corrected.

* p. 6 line 4 says "we" but only one of the two sources cited are from Agashe, et al. the other is from Wahl's group

This is now rephrased.

* p. 7 line 27 has a "^" for exponentiation

This is now corrected.

* p. 8 line 28 could you either have a math formula, or a verbal formula, and not mix the two?

We have now consistently used formulae.

* p. 9 line 6 replace "strain and ..." with "strain- and ..."

This is now corrected.

* Table 1 could use some uncertainties on bias estimates

We have now added confidence intervals.

* "indel bias" is ratio of indel to BPS? I have never heard it that way. I'm more interested in the ratio of deletions to insertions

Given the very small number of insertions and deletions observed, it would not be very useful in this case to think of their ratio.

* p. 11 lines 19, 22, there are some long "em" dashes in here where there should be short "en" dashes

This is now corrected.

* Fig 3 the color key does not apply to fig 3B, right?

It does apply to panel B (now Figure 4; see the key for panels B–F).

Reviewer #3

The study goal is very interesting and timely: building on previous experimental observations, the authors seek to test that E. coli strains that sample the space of one step mutations differently will evolve under different distribution of fitness effect (DFEs). To do so, authors use a set of mutation accumulation (MA) experiments that are seeded with ancestors that are either a wild type (K-12 MG1655) or different genotypes that have been constructed to represent an "isogenic" version of K-12 MG1655 but where several DNA repair genes have been knocked out. The rationale being here to manipulate as cleanly as possible, the spectrum of mutations and gain more precise knowledge about how different mutations class have different associated effects. Authors then go on to sequence a set of MA lines at times calibrated so relatively few mutations had time to accumulate and using whole genome sequencing the authors aim is to identify MA strains that differ from the ancestor by a single mutation in the genome. This is "on paper" a very elegant design as it is closest to the perfect thought experiment.

Thank you for the encouraging comments.

In practice ensuring that the strains are indeed truly isogenic and one single step away from the MA ancestor is a daunting task because of two crucial experimental challenges:

Challenge A: The collection of gene knockouts (ΔmutT, ΔmutH, ΔmutL, ΔmutS, Δnth, Δnei, ΔmutY) are obtained from another lineage (BW25113 strain) and uncontrolled "extra changes" in the genome of these isogenic strains can occur during the construction. The author acknowledge that in the discussion "despite attempts to minimize structural disruptions, some repair gene deletions may have caused regulatory changes in downstream genes, leading to strain-specific epistasis with new mutations", but no genomic data is presented to quantify and check to which extent the issue (see below for main comments) is problematic or minor.

Challenge B: Mutations accumulate stochastically in the genome so the number of mutations carried by a strain at a given timepoint is unknown, but in principle one has a collection of MA lines with 0, 1, 2 etc mutations (Poisson with the total genome-wide mutation rate U* nb generations). The authors employ whole genome sequencing (WGS) to detect new mutations that accumulated from the ancestor. The design only "works" if and only if the sequencing quality and the bioinformatic pipeline employed to call new variants has a very high sensitivity (so we do not "miss" new mutations that go undetected) and a very high sensitivity (so mutations calls are genuine mutations). Zero supplementary information is provided on that besides an overview of how many MA lines were sequenced in Table S1.

We appreciate these points, and agree that they were not adequately addressed in the manuscript. Please see below for a detailed response.

I have made a series of comments directly on the manuscript PDF, that have to do with presentation of figures etc. but my major issue at this stage is that zero data is presented to show that challenge A and B have been solved.

We have listed and responded to the PDF-marked comments at the end of this document.

To my knowledge, the only way to address challenge A is to sequence 4-5 clones of each isogenic strain used to seed the MA (currently only one is being sequenced according to table S1 when looking up the Anc(0) category) to make a proper comparison to the donor background of the knockouts and assess how many changes actually occurred in the background of the Isogenic strain. Sequencing more than 1 ancestor clone is also giving more confidence when detecting de novo mutations that these are not actually "segregating" in the ancestor.

We agree that this is an important point. However, note that segregating variants in the ancestor that are later identified as a single-step mutant during MA are a serious problem only if the variant affects the measured ancestral fitness (e.g., because it was segregating at high frequency, or was exceptionally beneficial or deleterious). In this case, we would not be able to accurately estimate the fitness effect of the mutation. This is more likely to be a problem for mutators with very high mutation rate (ΔmutH, ΔmutT, ΔmutL and ΔmutS), where it is practically impossible to avoid ancestral variants. However, as long as the segregating variants are rare, they are unlikely to significantly influence the ancestral growth rate measurement. Hence, our experimental protocols were designed to minimize the chances that ancestral variants would arise and would go undetected.

For the MA experiments, we isolated a single colony of each ancestor, suspended it in LB broth, and plated it to obtain as many colonies as were needed for each independent MA line. We used this same culture within 3-4 hours of growth to extract DNA for sequencing. For mutators with very high mutation rates, each MA block had its own ancestor (since each time cells are grown up from freezer stocks, there is a very high probability that new mutations will arise) whose sequence was used to subtract the ancestral mutations from offspring lines. For mutators with intermediate mutation rates and for WT, different MA blocks of a strain were started with the same ancestor. Ancestral mutation lists were cross-checked by confirming that all offspring MA lines seeded by a given ancestral clone showed the expected set of ancestral mutations at very high frequency. We sequenced ancestors at high depth (at least 40x) to allow detection of variants segregating at low frequency. For this, we relaxed the 80% allele frequency filter used to call evolved mutations. We either did not find any low-frequency alleles (e.g., typical for WT) or found them to be very rare (Supplementary Datasheet S1). Finally, we cross-checked that the single evolved mutations identified in mutators were not already segregating in their ancestor at low frequency.

We have now explained the MA and sequencing protocols in more detail in the Methods section. We have also added information on sequencing depth in Table S1, and added more information to the list of background mutations given in Supplementary Datasheet S1.

Similarly, to be convinced that challenge B is met, we need:
- information on the genomic coverage achieved for each MA line sequenced and some description of

the pipeline used and
- an assessment of the rate of false positive (spurious mutations that are not inherited in the next generation) and false negatives (mutations missed).
My own experience based on quite extensive in-house simulations of pipeline geared at detecting de novo mutations even in "simple" genome such as ref strains of E coli (data available upon request) is that achieving "only" 80-85% variant recall (eg 15-20% false negative) is quite common.

Thank you for these really useful suggestions. We have now added a new subsection at the beginning of the Results (“accuracy of mutation calling and fitness measurements”) to specifically discuss quality control measures for the sequencing and fitness measurements (also see the last paragraph of the Methods subsection “whole genome sequencing..”). Briefly, the key points are as follows.

We ensured that each sample had >18x depth (in most cases much higher than that; details are now given in Table S1). We used both Breseq and varscan to call mutations, and have added this information in the Methods section (subsection “whole genome sequencing…”, paragraph 2). We only called mutations that were supported by at least 4 reads per strand, and with >80% frequency. These stringent filters were designed to prevent false positives. We also measured the growth rate of a subset of single-mutation clones across two growth cycles; these are strongly positively correlated, suggesting high heritability and a low false positive rate (new Figure S6).

To check whether we had missed additional mutations in the single-mutation clones, we called mutations after relaxing the 80% frequency filter (but retaining the quality and read support filters). In most cases, few additional mutations were found, typically at low frequency (new Figure S3). Nonetheless, we tested whether these secondary mutations may have influenced our conclusions by affecting mutant fitness. For each strain, we constructed DFEs and estimated fb, fraction neutral and fd values after considering mutants with no additional mutation, those with a low-frequency second mutation, and those with two high-frequency mutations. The values did not change significantly (new Figure S9), suggesting weak effects of the secondary mutations.

To directly estimate the false negative rate, we used two mutations (a SNP and a 2-bp insertion) that were fixed in our WT ancestor relative to the NCBI reference strain, and should therefore have been detected in all our samples with 100% read support. Both mutations were observed in all evolved clones with a median frequency of 100% (SNP) and 93-96% (insertion) (new Figure S1).

Finally, we directly tested whether the results we report are robust to details of mutation calling, finding that the patterns do not change if we use clones with strictly 1 mutation, 1 additional low-frequency secondary mutation, or two mutations (Figure S9). Additionally, we tested the outcomes with reduced sample sizes, again finding only minor differences in the DFEs (Figure S10). This makes us more confident in our conclusions.

A very good and overall very honest test of the overall accuracy of the pipeline used here is to infer and report the number of mutations detected in each MA line at a given timepoint (Table S1), and besides saying just 346 lines are in the "more than 1" in ΔmutH and 100 have exactly 1, check whether these numbers are indeed Poisson distributed (the stopping rule was probably to accumulate enough lines with apparently 1 but the sampling is unbiased otherwise) in the expected ball park.

Thank you for this great suggestion. We now provide the exact number of mutations called for each sequenced timepoint (Supplementary datasheet S2), and details of a comparison against a Poisson distribution for each experimental MA block (Figure S2). The observed results are not significantly different from a Poisson expectation for any of the strains.

I am also wondering what the rate of detection of indels is for these genomes and wondering why zero mobile element activity is being called.

As described above, we estimated the false negative rate for an ancestral WT indel, and found that it was very low.

In other (unpublished) work from our lab we have observed dozens of IS element movements in populations evolved under selection, including in the same strains used here and with the same sequencing and analysis pipeline. Hence, we are confident that we can identify MGE movements. Baseline rates of MGE activity in *E coli* are on the order of 10^{-4} to 10^{-5} per genome per generation (Xia et al 2023 GBE, Haewook et al 2016 NAR). Given this, our mutators (evolved for a very short time) are expected to have 0-2 MGE movements and WT MA lines (evolved for many more generations) are expected to have 2-26 MGE movements. We confirmed (using Breseq analysis) that our single-mutation clones did not have any MGE mutations.

I also have two suggestions regarding the way the statistical analysis is being conducted on two points:
Instead of doing a flurry of pairwise comparison between strains and between mutation classes, it would be fruitful to first have a global linear model that decomposes the variation in fitness effects (aka the response variable) as a function of the molecular nature of the mutation (it could be Ts vs Tv and independently GC or AT biases or neutral) as well as codin or non coding etc.

We did conduct such an analysis, but we were worried that the factors were not completely independent and orthogonal, and given the strong mutational biases, sample sizes for some types of mutations per strain would be highly skewed. Hence, we chose to conduct specific analyses focusing on strains where direct comparisons were most meaningful with respect to our hypothesis.

The quantities of beneficial mutation flux Sb and load are only clearly described in the legend of Table S4 and there a important quantities that deserve to be properly introduced in the main text and given the way they are actually estimated:
Sb = fb x μ x genome size

One should quantify the statistical uncertainty surrounding these quantities (eg fb and μ) and overall on Sb.

We have now discussed the measurement of supply and load in the Methods section (subsection "estimating mutation rate…"), and have included uncertainty in all these quantities (Table 1, Table S4, Table S5).

In closing, I think the data presented here has tremendous potential, However, at this stage I cannot evaluate how reliable it is. In that light, analysing, and interpreting the differences detected for instance in Ts vsTv mutations and also the surprising lack of differences detected for other classes of mutations or the lack of effect of the initial level of adaptation is only possible if we first get confident that the points A and B above are not impacting massively the results reported.
I hope that my comments above and suggestions marked on the PDF will be received as constructive criticism and think that if the challenge A and B can be addressed, this is a uniquely interesting dataset / study for the community.

Thank you, these are indeed very useful suggestions that have improved our manuscript. We hope we have addressed all your concerns in our revision.

Points from the PDF marking (excluding comments that were already addressed above)

Main text file

1. Page 2, line 4: "An additional constraint emerges over the course of evolution" deleted

We have now deleted this sentence.

2. Page 3, line 6: "provides important information about the number and" replaced with "determines the"

Done.

3. Page 10, line 9: “Of the 94 mutations, 46 are second-, third-, or fourth-step mutations occurring in a given lineage, whose fitness effect was estimated with respect to the immediate ancestor (i.e., carrying first- or second-step mutations, respectively). Details are described in [14].” Comment: This is very hard to follow

We have now added more information here, and in Table S1. We hope that this is easier to follow.

4. Page 13, line 1: “Figure 1: Distribution of fitness effects”. Comment: Please report raw counts not frequencies

We cannot report raw counts here, because the DFE is corrected for selection bias, and this procedure requires the use of frequencies. However, raw fitness data are provided in Supplementary Datasheet S3.

5. Page 13, line 10: “account for experimental measurement error”. Comment: THat assumes that SE around fitness mesures doest not covary with growh rates (that is very unlikely)

We agree. However, we did not have the large number of measurements required to estimate the measurement error for each strain in each medium. Hence, we used the WT error estimates (with hundreds of repeated measurements) as a proxy.

6. Page 14, line 10: “s=0±0.025 to account for experimental measurement error)”. Comment: How come experimental error i glucose and LB is the same?

This is a fair point. We have now used a separate estimation of the experimental error in LB (which is higher than for glucose), re-fit the DFEs, and corrected all subsequent analyses (Figure 2, Figure 4A; Figures S7, S11, S12, S14; Tables S2, S4, S5). Some of the statistical details and values changed as a result, but the main conclusions remain unchanged.

7. Page 15, Figure 3, panel C: Comment: How is indel bias color coded? This is super hard to follow from the color figure legend below. Say for WT what does mean that base pair susbstitution are overwhelming?

Yes, this is the correct interpretation. We have now altered the figure legend to specify that bars are colored to reflect each strain’s mutation bias, and the shading reflects mutational class.

8. Page 16, line 26: “the fitness of the original deletion strains varied significantly in both media, it was not correlated with f_b (Figure S7”. Comment: I would test more globally with a general linear model the variation in fitness effect with original fitness level as a covariate (see general comments to author texts)

We are unsure about how the suggested analysis would be an improvement. In general, we have analysed data for the two environments separately throughout the manuscript, and we think this is useful with respect to understanding the outcomes in the two different environments.

9. Page 18, line 8: “paired Wilcoxon’s rank sum tests”. Comment: Why are paired tests used here? I do not understand why these should be paired tests given the design

Thank you for catching this error. We did not in fact use paired tests here. We have now corrected this in the figure legend.

10. Page 19, line 15: “as”. Comment: contrary to

We have now rephrased this sentence for clarity.

11. Page 20, line 7: “AT→GC mutations (maroon points) and GC→AT mutations (black points)”. Comment: what about GC / AT neutral mutations?

As noted in the figure legend, 9 mutations that did not alter GC content (i.e., GC→CG or AT→TA mutations) are not shown here.

12. Page 22, line 3: “suggesting a mechanism through which specific types of bias shifts may alter evolutionary outcomes”. Comment: I dont folloe which mechanism you are putting forward here.

We refer here to the enhanced sampling of beneficial mutations within poorly-explored mutational classes. We have now included an explanation (Discussion, paragraph 2).

13. Page 23, line 21: “(e.g., distinct protein-disruptive effects of GC→AT vs. AT→GC mutations)”. Comment: Distinguishing between AA replacement and syn and mild versus radical AA replacement should be done with this dataset

We have already shown some analyses, included in the supplementary material (Figure S13). More detailed analyses are planned for a subsequent paper.

14. Page 24, line 17: “may arise from complex interactions between these and/or other factors”. Comment: can you give a more specific example?

We are paraphrasing the authors of the cited reference here, and they do not unfortunately provide a specific example, likely because this is speculative.

15. Page 25, line 5: “Together, our results indicate that while the fitness consequences of each of these types of mutations is probably highly context-specific, such patterns are not observed at the genome-wide scale (i.e., the fitness effect of mutations varies across sites or genomic locations).” Comment: Given the large number of sinlge mutations measured here it might be nice to try and fit a linear model that looks jointly at different possible predictors of the type of mutations (eg syn versys mon syn and Ts versus Tv) to try and understand better how mu variation can be expplined in the actual variation in observed differences.

Please see our response to the main comments above.

Supplementary file

16. Figure S7: Comment: Fb also contains uncertainty please ass vertical +- SEs

We have now added this (this is now Figure S11).

17. Table S1, Row 10, column 3 & 4: Comment: Does not make sense are columns swapped?

No, the columns are not swapped. The confusion likely arises due to the larger number of single mutations noted, than the number of lines. This is because from some lineages, we used the 2nd- and 3rd- step mutations to quantify fitness effects relative to the immediate ancestor. We have now described this in more detail, as noted in our response to comment #3 above.

18. Table S2: “Benjamini-Hochberg statistic corrected”. Comment: how do you do the corrections? Across all tests or across a series of pairwise comparisons?

We do the tests across all comparisons. This is now noted in the legend.

19. Table S4: “Beneficial supply calculations”. Comment: uncertainty in fb mu and overall in Sb should be quanitfied

We have now done this.
