# Supplementary material for: Mutation bias alters the distribution of fitness effects of mutations
Source: PLoS Biol. 2025 Jul 14;23(7):e3003282. doi: 10.1371/journal.pbio.3003282 (PMC12273949; doi:10.1371/journal.pbio.3003282)
Supplement: Attachment — Submitted filename: ReviewerResponse.docx [file pbio.3003282.s054.docx]

**Editor’s comments**

a) Unfortunately reviewer #3 was unable to re-review; however, we have new comments from both of the others. Reviewer #1 signs off enthusiastically with no further requests. Reviewer #2 concedes that s/he was wrong on one of their previous concerns, but still wants further explanation on the second. S/he also wants you to address some significant presentational issues, and insists (with arguments why) that you include statistical results more prominently in the paper. Please address these remaining requests from reviewer #2.

Please see the point-by-point response to Reviewer 2’s concerns below.

b) Please address my Data Policy requests below; specifically, we need you to supply the numerical values underlying Figs 2, 3, 4, 5AB, 6ABCD, S1AB, S2, S3, S4, S5, S6, S7, S8, S9, S10, S11ABCD, S12 ABCD, S13ABC, S14, S15ABCDEF, either as a supplementary data file or as a permanent DOI’d deposition. I note that you already supplied the raw data in the existing supplementary data files, but we do also need the values directly underlying each Fig panel.

We have now provided separate files with data underlying all figures, bundled as an SI zip file.

c) Please cite the location of the data clearly in all relevant main and supplementary Figure legends, e.g. “The data underlying this Figure can be found in S1 Data” or “The data underlying this Figure can be found in <https://zenodo.org/records/XXXXXXXX>

We have now done this.

d) I note that you mention the reviewers ["and three reviewers (including Thomas Bataillon)"] in the Acknowledgements. While we appreciate the sentiment, this is against PLOS policy, so please could you remove it?

We have now removed this.

e) Please make any custom code available, either as a supplementary file or as part of your data deposition.

We have included custom code as supplementary files (S1-S7 scripts).

As you address these items, please take this last chance to review your reference list to ensure that it is complete and correct. If you have cited papers that have been retracted, please include the rationale for doing so in the manuscript text, or remove these references and replace them with relevant current references. Any changes to the reference list should be mentioned in the cover letter that accompanies your revised manuscript.

We have reviewed our reference list and have not made any changes.

In addition to these revisions, you will need to complete some formatting changes, which you will receive in a follow up email. A member of our team will be in touch with a set of requests shortly.

**Journal Requirements**

1) Your current Financial Disclosure states, “This work was funded by the DBT/Wellcome Trust India Alliance (Grant no. IA/S/23/2/506989 to DA), the National Centre for Biological Sciences (NCBS–TIFR) and the Department of Atomic Energy, Government of India (Project Identification No. RTI 4006 to DA), and the University Grants Commission, India (fellowship number 211610044747 to SP). The funders did not play any role in the study design, data collection and analysis, decision to publish, or preparation of the manuscript.”. 

However, your funding information on the submission form indicates that you did not receive funding. Please indicate by return email the full and correct funding information for your study and confirm the order in which funding contributions should appear. Please be sure to indicate whether the funders played any role in the study design, data collection and analysis, decision to publish, or preparation of the manuscript.

The current financial disclosure is correct and complete.

2) We ask that a manuscript source file is provided at Revision. Please upload your manuscript file as a .doc, .docx, .rtf or .tex.

This is done.

3) Please provide separate figure files in .tif or .eps format.

This is done.

4)  Please upload a copy of Figure [1 - 6] which you refer to in your text on your manuscript. Or, if the figure is no longer to be included as part of the submission please remove all reference to it within the text.

All Figure files are uploaded as .tif files.

5) We have noticed that you have uploaded Supporting Information files, but you have not included a list of legends. Please add a full list of legends for your Supporting Information files after the references list. 

This is done.

**Reviewer #1**

Firstly, I apologise for the late submission of this review. I continue to really like this paper and think it is an excellent contribution to the field - adding necessary experimental data to a largely theoretical area. The data are robust, and the experimental design is elegant and appropriate to the hypothesis. The authors have responded thoroughly to reviewer comments, and the manuscript is much improved as a result. I particularly appreciated the additional information provided on mutation calling and fitness estimation, which increased my confidence in the reliability of the results. The finding that bias reversal can increase the proportion of beneficial mutations is compelling and well supported. I congratulate the authors on a lovely piece of work. 

Thank you for your generous comments.

**Reviewer #2**

The authors have presented a revised submission, responding in a detailed way to 3 different reviews, each of which raised multiple points.  I appreciate all of their effort to improve the presentation of this very interesting and important study. 

My initial review included a critique that, upon consideration, was partly wrong. I argued that mutY should be excluded from any statistical test due to being used previously to argue the same kind of conclusion, and that the non-independence of mutants means that there are only 4 (or 3) data points, not 7. The first point remains valid and it is a simple matter to take this into account (by dropping the data from mutY but keeping mutT and the others). In careful statistical work, we do not purport to test a hypothesis that was proposed based on prior data in a way that includes the same prior data, because the hypothesis is not independent of that data. 

We again respectfully disagree with this point, for reasons described during the previous round of revision. We reiterate that the hypothesis tested here was based on simulations rather than on our previous data (which included a subset of the mutY and WT data reported here).

However, the second point is not valid. I argued that mutT and mutY were not independent, and likewise that mut S, L and H were not independent, due to hitting the same pathway. This is why I said there were only 4 data points and not 7. But this isn't really correct for the dependent variable, the DFE. The mutations that hit the same pathway are non-independent for the ti/tv bias (and the mutation spectrum generally), but the DFEs they generate are presumably largely independent because (presumably) they are mostly not sharing the same mutations. It would be useful for the authors to explain this if it is, in fact, true.

Thank you, we have now mentioned that each strain has unique mutations, in the Results section (page 16 line 9).

I still find problems with the presentation. The scatter-plots and correlations in Fig S3, in my opinion, are best suited to convey the main results. 

This is now Fig S11 (old Fig S3). As described in our response to comments for the first round of revisions, the relationship between fb and Tv bias is not the main focus of this work, and our study is underpowered to adequately test this correlation (as discussed in the main text). Hence, we do not want to make this a main figure.

I wish that the figures were not so confusing in their use of color. Figure 4 has 7 different color keys.

We appreciate the point about many colors in the figures, but given the complexity of the dataset (multiple strains, fitness effects, types of mutations), we do not see any other way to display the data. Note that in Fig 4, we have 3 (not 7) color keys, with labels indicating the relevant panel(s) for each key. We have also maintained consistency across figures to make it easier for readers to follow. Hence, we have retained the colors.

Fig 1 has a colored gradient and also a separate 3-color scheme. Fig 1 needs to present the logic of a prediction (and perhaps the logic of the theory that drives it): when the independent variable (mutation bias) is shifted, this induces (by biased depletion due to the effect of arrival bias) a shift in the dependent variable (DFE).  Having actual numbers for ti/tv bias is a distraction.

We have now changed the color gradient in Fig 1 to grasyscale. The numbers for ti/tv bias are helpful as an example of the magnitude of bias differences, so we have retained them. We agree that it would be nice to show the logic of biased depletion due to the effect of arrival bias, but we could not find a way to succinctly add this. We hope that the text, in conjunction with Fig 1 and legend, does clearly convey the logic and the prediction.

The authors repeatedly present results in the main text without providing a statistical finding, somethings doing this using words like "significant". They literally have a sentence that says our results support our predictions without specifying the precise substance of either the results or the predictions!  In some cases they have done tests, and in other cases they have made a qualitative comparison, e.g., p. 22 line 9 they are using a qualitative analysis in reaching conclusions about Fig. 4 "In contrast, no other axis of variation in mutation spectrum matched the pattern of variation in fb across strains (compare Figure 4A with Figures 4C-F). "

I want to stress that this is a quantitative empirical paper. The statistical results should be front and center, not something that one has to dig to find.  Saying "our results support out conclusions" without immediately backing that up with numbers is not satisfactory. 

All of the statistical analyses are either presented in the figures or main text next to statements describing the results. When there are many comparisons that are difficult to describe succinctly (e.g., for data shown in Figure 4), statistical outputs are provided in SI Tables, which are clearly referenced in the relevant Figure legend to make it easier to find.

For the sentence on p 22, your point is fair, thank you for noting this. We have now modified this sentence and reported correlations with other aspects of the mutation spectrum (they were not significant).

However, having said all that, the paper has been improved in response to the reviews and it does not need to be perfect. Ideally there will be other papers to follow from this group or from others.  So, I would recommend the paper for publication without further revision.

Thank you; we are glad that you find our work valuable.
